# Supplementary material for: Identification of Risk Factors in Patients with Recurrent Cystitis May Improve Individualized Management
Source: Diagnostics (Basel). 2025 Nov 14;15(22):2885. doi: 10.3390/diagnostics15222885 (PMC12651027; doi:10.3390/diagnostics15222885)

Supplementary Tables

Supplementary Table S1. Baseline demographics and medical history (including LUTIRE risk factors)

Parameter

**Demographic characteristics**	**Total**	**Sporadic acute cystitis (AC)**	**Recurrent cystitis (RC)**	**P-value (significance)**
Number of patients, n (%)	106 (100.0)	50 (47.2)	56 (52.8)	n.a.
Age, median (IQR)	36.5 (26.0-58.5)	36.0 (27.2-51.0)	37.5 (24.0-66.5)	0.219 (ns)
Weight in kg, median (IQR)	66.0 (57.0-74.8)	63.0 (56.0-70.0)	67.5 (60.0-75.0)	0.191 (ns)
Height in m, median (IQR)	1.6 (1.6-1.7)	1.6 (1.6-1.7)	1.7 (1.6-1.7)	0.054 (ns)
Body-mass index, median (IQR)	23.4 (21.3-27.5)	23.0 (21.3-27.2)	23.4 (21.4-27.7)	0.477 (ns)
Pregnancy, n (%)	2 (1.9)	1 (2.0)	1 (1.8)	1.000 (ns)
**Data from the medical history**	**Total**	**Sporadic acute cystitis (AC)**	**Recurrent cystitis (RC)**	**P-value (significance)**
At least one prior symptomatic episode of UTIs in the past 6	38 (35.8)	7 (14.0)	31 (55.4)	<0.001 (****)
At least one prior symptomatic episode of UTIs in the past 12	64 (60.4)	8 (16.0)	56 (100.0)	<0.001 (****)
Number of prior symptomatic episodes UTIs in the past 6	2.0 (1.0-5.0)	1.0 (0.0-1.0)	3.0 (2.0-5.0)	<0.001 (****)
Number of prior symptomatic episodes UTIs in the past 12	3.0 (0.0-12.0)	0.0 (0.0-0.0)	11.0 (5.0-40.0)	<0.001 (****)
Prior antimicrobial treatment for any reason in the past 3 months, n	41 (38.7)	15 (30.0)	26 (46.4)	0.125 (ns)
Prior antimicrobial treatment for any reason with a single agent in the past 3 months, n (%)	28 (26.4)	11 (22.0)	17 (30.4)	0.451 (ns)
Prior antimicrobial treatment for any reason with multiple agents in the past 3 months, n (%)	7 (6.6)	1 (2.0)	6 (10.7)	0.117 (ns)
No any prophylactic measure in the past 12 months, n (%)	55 (51.9)	37 (74.0)	18 (32.1)	<0.001 (****)
Single prophylactic measure in the past 12 months, n (%)	8 (7.5)	3 (6.0)	5 (8.9)	0.720 (ns)
Multiple prophylactic measures in the past 12 months, n (%)	43 (40.6)	10 (20.0)	33 (58.9)	<0.001 (***)
Duration of a current acute episode in days, median (IQR)	3.5 (2.0-6.0)	4.0 (3.0-5.0)	3.0 (2.0-6.5)	0.377 (ns)
Current episode was attempted to treat, n (%)	21 (19.8)	9 (18.0)	12 (21.4)	0.843 (ns)
**Risk factors for recurrent UTIs from the medical history according to the LUTIRE nomogram**	**Total**	**Sporadic acute cystitis (AC)**	**Recurrent cystitis (RC)**	**P-value (significance)**
Single sexual partner in the past year, n (%)	92 (86.8)	40 (80.0)	52 (92.9)	0.096 (ns)
Two sexual partners in the past year, n (%)	7 (6.6)	5 (10.0)	2 (3.6)	0.251 (ns)

Three or more sexual partners in the past year, n (%)	7 (6.6)	5 (10.0)	2 (3.6)	0.251 (ns)
Normal bowel function, n (%)	82 (77.4)	43 (86.0)	39 (69.6)	0.076 (ns)
Predisposed to chronic diarrhea, n (%)	6 (5.7)	4 (8.0)	2 (3.6)	0.418 (ns)
Predisposed to chronic obstipation, n (%)	18 (17.0)	3 (6.0)	15 (26.8)	0.005 (**)
Known Gram-positive uropathogen isolated at the last acute episode, n (%)	8 (7.5)	2 (4.0)	6 (10.7)	0.277 (ns)
Known Gram-negative uropathogen isolated at the last acute episode, n (%)	25 (23.6)	4 (8.0)	21 (37.5)	<0.001 (***)
No known uropathogen in the past, n (%)	73 (68.9)	44 (88.0)	29 (51.8)	<0.001 (***)
Premenopausal hormonal status, n (%)	72 (67.9)	37 (74.0)	35 (62.5)	0.290 (ns)
Postmenopausal hormonal status, n (%)	34 (32.1)	13 (26.0)	21 (37.5)	0.290 (ns)
Up to 2 acute episodes per year, n (%)	52 (49.1)	50 (100.0)	2 (3.6)	<0.001 (****)
Three or more acute episodes per year, n (%)	54 (50.9)	0 (0.0)	54 (96.4)	<0.001 (****)
Any antimicrobial therapy due to ASB in the past, n (%)	28 (26.4)	9 (18.0)	19 (33.9)	0.102 (ns)
No antimicrobial therapy due to ASB in the past, n (%)	78 (73.6)	41 (82.0)	37 (66.1)	0.102 (ns)
Probability of recurrence according to LUTIRE nomogram, median (IQR)	0.30 (0.20-0.40)	0.20 (0.20-0.30)	0.40 (0.30-0.50)	<0.001 (****)

Supplementary Table S2. Clinical examination findings and ORENUC risk categories at baseline

Parameter

Risk factors (abnormalities) of a more severe outcome according to the ORENUC-System	Total	Sporadic acute cystitis (AC)	Recurrent cystitis (RC)	P-value (significance)
O - No known risk factor, n (%)	56 (52.8)	37 (74.0)	19 (33.9)	<0.001 (****)
R - Risk factors for recurrent UTIs, but no risk of more severe outcome,	32 (30.2)	9 (18.0)	23 (41.1)	0.018 (*)
E - Extra urogenital risk factors with risk of more severe outcome, n (%)	6 (5.7)	0 (0.0)	6 (10.7)	0.028 (*)
N - Nephropathic diseases with risk of more severe outcome, n (%)	1 (0.9)	1 (2.0)	0 (0.0)	0.472 (ns)
U - Urological risk factors with risk of more severe outcome, which can be resolved during therapy, n (%)	8 (7.5)	3 (6.0)	5 (8.9)	0.720 (ns)
C - Permanent urinary catheter and non-resolvable urological risk factors with risk of more severe	0 (0.0)	0 (0.0)	0 (0.0)	1.000 (ns)
More than one risk factor, n (%)	3 (2.8)	0 (0.0)	3 (5.4)	0.245 (ns)
Revealed abnormalities of the	9 (8.5)	4 (8.0)	5 (8.9)	1.000 (ns)
**Assessment of the presence of additional conditions at the baseline visit (ACSS)**	**Total**	**Sporadic acute cystitis (AC)**	**Recurrent cystitis (RC)**	**P-value (significance)**
Menstruations, n (%)	12 (11.3)	7 (14.0)	5 (8.9)	0.542 (ns)
Premenstrual symptoms, n (%)	9 (8.5)	6 (12.0)	3 (5.4)	0.301 (ns)
Symptoms of menopause, n (%)	9 (8.5)	8 (16.0)	1 (1.8)	0.012 (*)
Pregnancy, n (%)	2 (1.9)	1 (2.0)	1 (1.8)	1.000 (ns)
Sugar diabetes, n (%)	9 (8.5)	4 (8.0)	5 (8.9)	1.000 (ns)

Supplementary Table S3. Baseline symptom severity (ACSS) and health status (EQ-5D-3L) Parameter

Assessment of the typical symptoms at the baseline visit (ACSS)	Total	Sporadic acute cystitis (AC)	Recurrent cystitis (RC)	P-value (significance)
**Frequent urination, n (%)**	**96 (90.6)**	**46 (92.0)**	**50 (89.3)**	**0.885 (ns)**
Mild, n (%)	21 (19.8)	11 (22.0)	10 (17.9)	0.772 (ns)
Moderate, n (%)	40 (37.7)	19 (38.0)	21 (37.5)	1.000 (ns)
Severe, (n%)	35 (33.0)	16 (32.0)	19 (33.9)	0.997 (ns)
**Urgent urination, (n%)**	**96 (90.6)**	**45 (90.0)**	**51 (91.1)**	**1.000 (ns)**
Mild, (n%)	18 (17.0)	10 (20.0)	8 (14.3)	0.601 (ns)
Moderate, (n%)	39 (36.8)	18 (36.0)	21 (37.5)	1.000 (ns)
Severe, (n%)	39 (36.8)	17 (34.0)	22 (39.3)	0.718 (ns)
**Painful urination, (n%)**	**92 (86.8)**	**44 (88.0)**	**48 (85.7)**	**0.952 (ns)**
Mild, (n%)	15 (14.2)	7 (14.0)	8 (14.3)	1.000 (ns)
Moderate, (n%)	32 (30.2)	19 (38.0)	13 (23.2)	0.149 (ns)
Severe, (n%)	45 (42.5)	18 (36.0)	27 (48.2)	0.283 (ns)
**Sense of incomplete bladder emptying, (n%)**	**82 (77.4)**	**36 (72.0)**	**46 (82.1)**	**0.311 (ns)**
Mild, (n%)	20 (18.9)	5 (10.0)	15 (26.8)	0.050 (ns)
Moderate, (n%)	29 (27.4)	19 (38.0)	10 (17.9)	0.035 (*)
Severe, (n%)	33 (31.1)	12 (24.0)	21 (37.5)	0.198 (ns)
**Suprapubic pain, (n%)**	**84 (79.2)**	**39 (78.0)**	**45 (80.4)**	**0.953 (ns)**
Mild, (n%)	16 (15.1)	8 (16.0)	8 (14.3)	1.000 (ns)
Moderate, (n%)	28 (26.4)	14 (28.0)	14 (25.0)	0.897 (ns)
Severe, (n%)	40 (37.7)	17 (34.0)	23 (41.1)	0.583 (ns)
**Visible blood in urine, (n%)**	**29 (27.4)**	**17 (34.0)**	**12 (21.4)**	**0.218 (ns)**
Mild, (n%)	12 (11.3)	6 (12.0)	6 (10.7)	1.000 (ns)
Moderate, (n%)	11 (10.4)	8 (16.0)	3 (5.4)	0.110 (ns)
Severe, (n%)	6 (5.7)	3 (6.0)	3 (5.4)	1.000 (ns)
**Assessment of the differential symptoms at the baseline visit (ACSS)**	**Total**	**Sporadic acute cystitis (AC)**	**Recurrent cystitis (RC)**	**P-value (significance)**
**Flank pain, (n%)**	**50 (47.2)**	**27 (54.0)**	**23 (41.1)**	**0.256 (ns)**
Mild, (n%)	22 (20.8)	9 (18.0)	13 (23.2)	0.674 (ns)
Moderate, (n%)	15 (14.2)	8 (16.0)	7 (12.5)	0.813 (ns)
Severe, (n%)	13 (12.3)	10 (20.0)	3 (5.4)	0.035 (*)
**Abnormal vaginal discharge, (n%)**	**29 (27.4)**	**16 (32.0)**	**13 (23.2)**	**0.427 (ns)**
Mild, (n%)	19 (17.9)	12 (24.0)	7 (12.5)	0.198 (ns)
Moderate, (n%)	4 (3.8)	1 (2.0)	3 (5.4)	0.620 (ns)
Severe, (n%)	6 (5.7)	3 (6.0)	3 (5.4)	1.000 (ns)
**Urethral discharge, (n%)**	**4 (3.8)**	**3 (6.0)**	**1 (1.8)**	**0.341 (ns)**
Mild, (n%)	1 (0.9)	1 (2.0)	0 (0.0)	0.472 (ns)
Moderate, (n%)	2 (1.9)	2 (4.0)	0 (0.0)	0.220 (ns)
Severe, (n%)	1 (0.9)	0 (0.0)	1 (1.8)	1.000 (ns)
**Sense of fever/chills, (n%)**	**21 (19.8)**	**11 (22.0)**	**10 (17.9)**	**0.772 (ns)**
Mild, (n%)	10 (9.4)	5 (10.0)	5 (8.9)	1.000 (ns)
Moderate, (n%)	6 (5.7)	3 (6.0)	3 (5.4)	1.000 (ns)
Severe, (n%)	5 (4.7)	3 (6.0)	2 (3.6)	0.665 (ns)

**Assessment of the quality of life at the baseline visit (ACSS)**	**Total**	**Sporadic acute cystitis (AC)**	**Recurrent cystitis (RC)**	**P-value (significance)**
**General discomfort, (n%)**	**104 (98.1)**	**49 (98.0)**	**55 (98.2)**	**1.000 (ns)**
Mild, (n%)	23 (21.7)	13 (26.0)	10 (17.9)	0.436 (ns)
Moderate, (n%)	60 (56.6)	28 (56.0)	32 (57.1)	1.000 (ns)
Severe, (n%)	21 (19.8)	8 (16.0)	13 (23.2)	0.493 (ns)
Impact on everyday life/activities, (n%)	99 (93.4)	46 (92.0)	53 (94.6)	0.877 (ns)
Mild, (n%)	40 (37.7)	20 (40.0)	20 (35.7)	0.800 (ns)
Moderate, (n%)	41 (38.7)	23 (46.0)	18 (32.1)	0.207 (ns)
Severe, (n%)	18 (17.0)	3 (6.0)	15 (26.8)	0.005 (**)
Impact on social life/activities, (n%)	93 (87.7)	43 (86.0)	50 (89.3)	0.827 (ns)
Mild, (n%)	34 (32.1)	18 (36.0)	16 (28.6)	0.542 (ns)
Moderate, (n%)	39 (36.8)	18 (36.0)	21 (37.5)	1.000 (ns)
Severe, (n%)	20 (18.9)	7 (14.0)	13 (23.2)	0.336 (ns)
**Summary scores of the ACSS domains at the baseline visit**	**Total**	**Sporadic acute cystitis (AC)**	**Recurrent cystitis (RC)**	**P-value (significance)**
Summary score of the 'Typical' domain, median (IQR)	9.5 (8.0-12.0)	9.0 (8.0-12.0)	10.0 (8.0-12.2)	0.730 (ns)
Summary score of the 'Differential' domain, median (IQR)	1.0 (0.0-3.0)	2.0 (0.0-3.0)	1.0 (0.0-2.0)	0.445 (ns)
Summary score of the 'QoL' domain, median (IQR)	5.0 (4.0-7.0)	5.0 (3.0-6.0)	5.5 (4.0-7.2)	0.445 (ns)
**Assessment of the general health condition the moment of the current admission (EQ-5D-3L)**	**Total**	**Sporadic acute cystitis (AC)**	**Recurrent cystitis (RC)**	**P-value (significance)**
**Mobility**				
No problems in walking about, n (%)	60 (56.6)	33 (66.0)	27 (48.2)	0.099 (ns)
Some problems in walking about, n (%)	33 (31.1)	13 (26.0)	20 (35.7)	0.385 (ns)
Confined to bed, n (%)	3 (2.8)	0 (0.0)	3 (5.4)	0.245 (ns)
Missing values, n (%)	10 (9.4)	4 (8.0)	6 (10.7)	0.746 (ns)
**Self-care**				
No problems with self-care, n (%)	86 (81.1)	42 (84.0)	44 (78.6)	0.642 (ns)
Some problems washing or dressing, n (%)	10 (9.4)	4 (8.0)	6 (10.7)	0.746 (ns)
Unable to wash or dress, n (%)	0 (0.0)	0 (0.0)	0 (0.0)	n.a.
Missing values, n (%)	10 (9.4)	4 (8.0)	6 (10.7)	0.746 (ns)
**Usual activities**				
No problems with performing usual activities, n (%)	59 (55.7)	35 (70.0)	24 (42.9)	0.009 (**)
Some problems with performing usual activities, n (%)	30 (28.3)	10 (20.0)	20 (35.7)	0.115 (ns)
Unable to perform usual activities, n (%)	7 (6.6)	1 (2.0)	6 (10.7)	0.117 (ns)
Missing values, n (%)	10 (9.4)	4 (8.0)	6 (10.7)	0.746 (ns)
**Pain or discomfort**				
No pain or discomfort, n (%)	27 (25.5)	14 (28.0)	13 (23.2)	0.733 (ns)
Moderate pain or discomfort, n (%)	51 (48.1)	23 (46.0)	28 (50.0)	0.828 (ns)
Extreme pain or discomfort, n (%)	18 (17.0)	9 (18.0)	9 (16.1)	0.996 (ns)
Missing values, n (%)	10 (9.4)	4 (8.0)	6 (10.7)	0.746 (ns)
**Anxiety/Depression**				

Not anxious or depressed, n (%)	62 (58.5)	33 (66.0)	29 (51.8)	0.199 (ns)
Moderately anxious or depressed, n (%)	27 (25.5)	13 (26.0)	14 (25.0)	1.000 (ns)
Extremely anxious or depressed, n (%)	7 (6.6)	0 (0.0)	7 (12.5)	0.014 (*)
Missing values, n (%)	10 (9.4)	4 (8.0)	6 (10.7)	0.746 (ns)
**General health status, median (IQR)**	60.0 (30.0-80.0)	60.0 (30.0-81.2)	60.0 (32.0-80.0)	0.820 (ns)

Supplementary Table S4. Urinalysis and urine culture findings at baseline, including antimicrobial Parameter

Results of the urine tests at the baseline visit	Total	Sporadic acute cystitis (AC)	Recurrent cystitis (RC)	P-value (significance)
Nitrite positive, n (%)	44 (41.5)	20 (40.0)	24 (42.9)	0.920 (ns)
Leucocyte-esterase test as Negative, n (%)	12 (11.3)	2 (4.0)	10 (17.9)	0.032 (*)
Leucocyte-esterase test as Trace (±), n (%)	5 (4.7)	1 (2.0)	4 (7.1)	0.367 (ns)
Leucocyte-esterase test as Small (1+), n (%)	12 (11.3)	4 (8.0)	8 (14.3)	0.369 (ns)
Leucocyte-esterase test as Moderate (2+), n (%)	28 (26.4)	18 (36.0)	10 (17.9)	0.001 (**)
Leucocyte-esterase test as Large (3+), n (%)	49 (46.2)	25 (50.0)	24 (42.9)	1.000 (ns)
Pyuria*, n (%)	77 (72.6)	43 (86.0)	34 (60.7)	0.007 (*)
Both nitrite AND leucocyte-esterase tests are negative, n (%)	8 (7.5)	1 (2.0)	7 (12.5)	0.063 (ns)
Both nitrite AND leucocyte-esterase tests are positive, n (%)	36 (34.0)	18 (36.0)	18 (32.1)	0.831 (ns)
RBC in urine as Negative, n(%)	79 (74.5)	40 (80.0)	39 (69.6)	0.318 (ns)
RBC in urine as Trace (±), n(%)	1 (0.9)	1 (2.0)	0 (0.0)	0.472 (ns)
RBC in urine as Small (1+), n(%)	1 (0.9)	1 (2.0)	0 (0.0)	0.472 (ns)
RBC in urine as Moderate (2+), n(%)	8 (7.5)	0 (0.0)	8 (14.3)	0.006 (**)
RBC in urine as Large (3+), n(%)	17 (16.0)	8 (16.0)	9 (16.1)	1.000 (ns)
Proteine in urine as Negative, n(%)	89 (84.0)	44 (88.0)	45 (80.4)	0.421 (ns)
Proteine in urine as Trace (±), n(%)	10 (9.4)	1 (2.0)	9 (16.1)	0.018 (*)
Proteine in urine as Small (1+), n(%)	2 (1.9)	1 (2.0)	1 (1.8)	1.000 (ns)
Proteine in urine as Moderate (2+), n(%)	3 (2.8)	2 (4.0)	1 (1.8)	0.601 (ns)
Proteine in urine as Large (3+), n(%)	2 (1.9)	2 (4.0)	0 (0.0)	0.220 (ns)
**Results of the microbiological investigation of urine samples of the current admission (urine culture)**	**Total**	**Sporadic acute cystitis (AC)**	**Recurrent cystitis (RC)**	**P-value (significance)**
Positive urine culture (≥10^3), n (%)	87 (82.1)	33 (66.0)	54 (96.4)	<0.001 (***)
Positive urine culture AND positive pyuria, n (%)	71 (67.0)	30 (60.0)	41 (73.2)	0.216 (ns)
Single uropathogen, n (%)	63 (59.4)	30 (60.0)	33 (58.9)	1.000 (ns)
Multiple uropathogens, n (%)	24 (22.6)	3 (6.0)	21 (37.5)	<0.001 (***)
**First pathogen**				
Gram positive species, n (%)	15 (17.2)	3 (9.1)	12 (22.2)	0.306 (ns)
Enterococcus sp., n (%)	4 (4.6)	0 (0.0)	4 (7.4)	0.293 (ns)
Gardnerella vaginalis, n (%)	1 (1.1)	0 (0.0)	1 (1.9)	1.000 (ns)
Lactobacillus sp., n (%)	1 (1.1)	0 (0.0)	1 (1.9)	1.000 (ns)
Staphylococcus aureus, n (%)	1 (1.1)	1 (3.0)	0 (0.0)	0.379 (ns)
Staphylococcus saprophyticus, n (%)	4 (4.6)	2 (6.1)	2 (3.7)	0.632 (ns)
Other coagulase-negative Staphylococci, n (%)	3 (3.4)	0 (0.0)	3 (5.6)	0.285 (ns)
Other Gram+ bacteria, n (%)	1 (1.1)	0 (0.0)	1 (1.9)	1.000 (ns)
Gram negative species, n (%)	65 (74.7)	25 (75.8)	40 (74.1)	0.985 (ns)

Escherichia coli, n (%)	55 (63.2)	22 (66.7)	33 (61.1)	0.770 (ns)
Klebsiella sp., n (%)	8 (9.2)	3 (9.1)	5 (9.3)	1.000 (ns)
Pseudomonas aeruginosa, n (%)	2 (2.3)	0 (0.0)	2 (3.7)	0.524 (ns)
Mixed flora (unknown Gram stain), n (%)	7 (8.0)	5 (15.2)	2 (3.7)	0.099 (ns)
**CFU of the first pathogen**				
CFU>=10^3, n (%)	6 (6.9)	4 (12.1)	2 (3.7)	0.195 (ns)
CFU>=10^4, n (%)	27 (31.0)	12 (36.4)	15 (27.8)	0.548 (ns)
CFU>=10^5, n (%)	20 (23.0)	6 (18.2)	14 (25.9)	0.568 (ns)
CFU>=10^6, n (%)	34 (39.1)	11 (33.3)	23 (42.6)	0.527 (ns)
**Second pathogen**				
Gram positive species, n (%)	15 (62.5)	1 (33.3)	14 (66.7)	0.533 (ns)
Aerococcus urinae, n (%)	2 (8.3)	0 (0.0)	2 (9.5)	1.000 (ns)
Enterococcus sp., n (%)	4 (16.7)	0 (0.0)	4 (19.0)	1.000 (ns)
Group B beta-haemolytic Streptococci, n (%)	4 (16.7)	1 (33.3)	3 (14.3)	0.437 (ns)
Lactobacillus sp., n (%)	1 (4.2)	0 (0.0)	1 (4.8)	1.000 (ns)
Staphylococcus epidermidis, n (%)	1 (4.2)	0 (0.0)	1 (4.8)	1.000 (ns)
Mixed flora (Gram positive), n (%)	3 (12.5)	0 (0.0)	3 (14.3)	1.000 (ns)
Gram negative species, n (%)	7 (29.2)	1 (33.3)	6 (28.6)	1.000 (ns)
Citrobacter sp., n (%)	1 (4.2)	0 (0.0)	1 (4.8)	1.000 (ns)
Enterobacter sp., n (%)	1 (4.2)	0 (0.0)	1 (4.8)	1.000 (ns)
Klebsiella sp., n (%)	1 (4.2)	0 (0.0)	1 (4.8)	1.000 (ns)
Morganella sp., n (%)	1 (4.2)	0 (0.0)	1 (4.8)	1.000 (ns)
Proteus sp., n (%)	2 (8.3)	0 (0.0)	2 (9.5)	1.000 (ns)
Other Enterobacteriaceae, n (%)	1 (4.2)	1 (33.3)	0 (0.0)	0.125 (ns)
Mixed flora (unknown Gram stain), n (%)	2 (8.3)	1 (33.3)	1 (4.8)	0.239 (ns)
**CFU of the second pathogen**				
CFU>=10^3, n (%)	7 (29.2)	2 (66.7)	5 (23.8)	0.267 (ns)
CFU>=10^4, n (%)	6 (25.0)	1 (33.3)	5 (23.8)	1.000 (ns)
CFU>=10^5, n (%)	7 (29.2)	0 (0.0)	7 (33.3)	0.693 (ns)
CFU>=10^6, n (%)	4 (16.7)	0 (0.0)	4 (19.0)	1.000 (ns)
Non-susceptibility rates of *E. coli* isolates to different classes of antimicrobial agents				
Aminoglycosides, n (%)	1 (1.0)	1 (2.0)	0 (0.0)	1.000 (ns)
1-st gen. Cephalosporins, n (%)	3 (3.1)	3 (5.9)	0 (0.0)	0.244 (ns)
2-nd gen. Cephalosporins, n (%)	28 (28.9)	9 (17.6)	19 (41.3)	0.019 (*)
3-rd gen. Cephalosporins, n (%)	5 (5.2)	5 (9.8)	0 (0.0)	0.058 (ns)
5-th gen. Cephalosporins, n (%)	1 (1.0)	1 (2.0)	0 (0.0)	1.000 (ns)
Fosfomycin, n (%)	1 (1.0)	1 (2.0)	0 (0.0)	1.000 (ns)
Fluoroquinolones, n (%)	12 (12.4)	8 (15.7)	4 (8.7)	0.364 (ns)
Penicillins, n (%)	22 (22.7)	10 (19.6)	12 (26.1)	0.604 (ns)
Penicillins + β-lactamase inhibitors, n (%)	14 (14.4)	7 (13.7)	7 (15.2)	1.000 (ns)
Tetracyclines, n (%)	2 (2.1)	2 (3.9)	0 (0.0)	0.496 (ns)

Trimethoprim, n (%)	8 (8.2)	4 (7.8)	4 (8.7)	1.000 (ns)

*Pyuria is defined as lecocyte-esterase test results as Moderate (2+) and Large (3+)

Supplementary Table S5. Relative risk (RR) estimates for parameters associated with AC vs RC (95% CI)

Parameter	RR (95% CI)	P-value (Significance)
Predisposed to chronic obstipation	1.79 (1.32; 2.43)	0.004
E - Extra urogenital risk factors	2.00 (1.64; 2.43)	0.050
Moderate sense of incomplete bladder emptying	0.58 (0.34; 0.98)	0.020
Severe flank pain	0.40 (0.15; 1.11)	0.022
Severe impact of symptoms on everyday life/activities	1.79 (1.32; 2.43)	0.004
Symptoms of menopause	0.20 (0.03, 1.25)	0.023
No problems with performing usual activities	0.58 (0.40, 0.84)	0.005
Extremely expressed anxiety or depression	2.07 (1.67, 2.57)	0.025
Leucocyte-esterase test as "Negative"	1.70 (1.23, 2.36)	0.025
Leucocyte-esterase test as "Moderate (2+)"	0.61 (0.36, 1.03)	0.034
Pyuria	0.58 (0.42, 0.81)	0.004
RBC in urine as "Moderate (2+)"	2.04 (1.67, 2.50)	0.016
Proteine in urine as "Trace (±)"	1.84 (1.37, 2.46)	0.032
Positive urine culture (≥10^3)*	7.43 (0.52, 106.13)	0.032
Multiple uropathogens	1.80 (1.35, 2.40)	<0.001
Non-susceptibility rates of E. coli isolates to 2-nd gen. Cephalosporins	1.48 (0.89, 2.45)	0.109

*Note: Haldane-Anscombe correction for "zero-cells" https://doi.org/10.1002/jrsm.1460

Supplementary Table S6. Candidate predictors selected for uni- and multivariable logistic regression modeling

Parameter

**Risk factors for recurrent UTIs from the medical history according to the LUTIRE nomogram**	**Total**	**Sporadic acute cystitis (AC)**	**Recurrent cystitis (RC)**	**P-value (significance)**
Predisposed to chronic obstipation, n (%)	18 (17.0)	3 (6.0)	15 (26.8)	0.005 (**)
**Risk factors (abnormalities) of a more severe outcome according to the ORENUC-System**	**Total**	**Sporadic acute cystitis (AC)**	**Recurrent cystitis (RC)**	**P-value (significance)**
E - Extra urogenital risk factors with risk of more severe outcome, n (%)	6 (5.7)	0 (0.0)	6 (10.7)	0.028 (*)
**Symptoms and the quality of life according to the ACSS at the baseline visit**	**Total**	**Sporadic acute cystitis (AC)**	**Recurrent cystitis (RC)**	**P-value (significance)**
Moderate sense of incomplete bladder emptying, n (%)	29 (27.4)	19 (38.0)	10 (17.9)	0.035 (*)
Severe flank pain, n (%)	13 (12.3)	10 (20.0)	3 (5.4)	0.035 (*)
Severe impact of symptoms on everyday life/activities, n (%)	18 (17.0)	3 (6.0)	15 (26.8)	0.005 (**)
Symptoms of menopause, n (%)	9 (8.5)	8 (16.0)	1 (1.8)	0.012 (*)
**Assessment of the general health condition at the baseline visit (EQ-5D-3L)**	**Total**	**Sporadic acute cystitis (AC)**	**Recurrent cystitis (RC)**	**P-value (significance)**
No problems with performing usual activities, n	59 (55.7)	35 (70.0)	24 (42.9)	0.009 (**)
Extremely expressed anxiety or depression, n (%)	7 (6.6)	0 (0.0)	7 (12.5)	0.014 (*)
**Results of the urine tests at the baseline visit**	**Total**	**Sporadic acute cystitis (AC)**	**Recurrent cystitis (RC)**	**P-value (significance)**
Negative leucocyte-esterase test, n (%)	12 (11.3)	2 (4.0)	10 (17.9)	0.032 (*)
Leucocyte-esterase test as "Moderate (2+)", n	28 (26.4)	18 (36.0)	10 (17.9)	0.001 (**)
Pyuria*, n (%)	77 (72.6)	43 (86.0)	34 (60.7)	0.007 (*)
RBC in urine as Moderate (2+), n(%)	8 (7.5)	0 (0.0)	8 (14.3)	0.006 (**)
Proteine in urine as Trace (±), n(%)	10 (9.4)	1 (2.0)	9 (16.1)	0.018 (*)
**Results of the microbiological investigation of urine samples at the baseline visit (urine culture)**	**Total**	**Sporadic acute cystitis (AC)**	**Recurrent cystitis (RC)**	**P-value (significance)**
Positive urine culture (≥10^3), n (%)	87 (82.1)	33 (66.0)	54 (96.4)	<0.001 (***)
Multiple uropathogens, n (%)	24 (22.6)	3 (6.0)	21 (37.5)	<0.001 (***)
Non-susceptibility rates of E. coli isolates to 2-nd gen. Cephalosporins, n (%)	28 (28.9)	9 (17.6)	19 (41.3)	0.019 (*)

Multivariate Logistic Regression Model I

Includes Predisposition to chronic obstipation (LUTIRE); Severe impact of symptoms on everyday life/activities (ACSS); Multiple uropathogens in urine culture (MIBI); Proteine in urine as “Trace (±)” (Dipstick); Pyuria, defined as “Moderate (2+)” and “Large (3+)” results of leucocyte-esterase test (Dipstick)

Summary of the Final Logistic Regression Model I

Parameter	Estimate	Std. Error	z value	P-value
(Intercept)	0.052	0.512	0.101	0.919
Predisposed to chronic obstipation	1.906	0.757	2.517	0.012
Severe impact of symptoms on everyday life/activities	1.862	0.764	2.438	0.015
Multiple uropathogens	2.529	0.722	3.504	0.000
Proteine in urine as Trace	2.498	1.162	2.150	0.032
Pyuria	-1.476	0.582	-2.534	0.011

ROC Coordinates of the Final Logistic Regression Model I

Measure	Value	CI95
threshold	0.35	(0.35–0.87)
sensitivity	0.86	(0.50–0.93)
specificity	0.70	(0.62–1.00)
youden	1.56	(1.44–1.71)
ppv	0.76	(0.71–1.00)
npv	0.81	(0.64–0.91)

Confusion Matrix and Statistics

```
      Reference
```

Prediction 0 1 0 35 8 1 15 48

```
          Accuracy : 0.783
            95% CI : (0.6924, 0.8572)
No Information Rate : 0.5283
P-Value [Acc > NIR] : 5e-08

             Kappa : 0.5614
```

Mcnemar’s Test P-Value : 0.2109

```
       Sensitivity : 0.8571
       Specificity : 0.7000
    Pos Pred Value : 0.7619
    Neg Pred Value : 0.8140
        Prevalence : 0.5283
    Detection Rate : 0.4528
```

Detection Prevalence : 0.5943
Balanced Accuracy : 0.7786

```
   'Positive' Class : 1
```

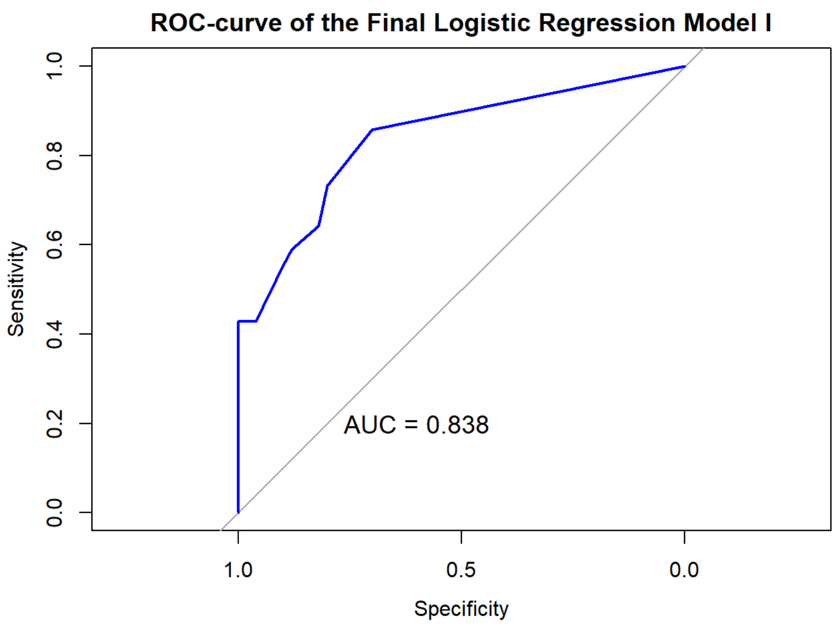

Multivariate Logistic Regression Model II

Includes Predisposition to chronic obstipation (LUTIRE); Severe impact of symptoms on everyday life/activities (ACSS); Multiple uropathogens in urine culture (MIBI); Pyuria, defined as “Moderate (2+)” and “Large (3+)” results of leucocyte-esterase test (Dipstick)

Summary of the Final Logistic Regression Model II

Parameter	Estimate	Std. Error	z value	P-value
(Intercept)	0.277	0.491	0.564	0.573
Predisposed to chronic obstipation	1.731	0.743	2.330	0.020
Severe impact of symptoms on everyday life/activities	1.868	0.742	2.517	0.012
Multiple uropathogens	2.434	0.706	3.448	0.001
Pyuria	-1.488	0.562	-2.645	0.008

ROC Coordinates of the Final Logistic Regression Model II

Measure	Value	CI95
threshold	0.40	(0.40–0.64)
sensitivity	0.82	(0.57–0.91)
specificity	0.72	(0.62–0.94)
youden	1.54	(1.40–1.70)
ppv	0.77	(0.71–0.93)
npv	0.78	(0.65–0.88)

Confusion Matrix and Statistics

```
      Reference
```

Prediction 0 1 0 36 10 1 14 46

```
          Accuracy : 0.7736
            95% CI : (0.6821, 0.8492)
No Information Rate : 0.5283
P-Value [Acc > NIR] : 1.581e-07

             Kappa : 0.5438
```

Mcnemar’s Test P-Value : 0.5403

```
       Sensitivity : 0.8214
       Specificity : 0.7200
    Pos Pred Value : 0.7667
    Neg Pred Value : 0.7826
        Prevalence : 0.5283
    Detection Rate : 0.4340
```

Detection Prevalence : 0.5660
Balanced Accuracy : 0.7707

```
  'Positive' Class : 1
```

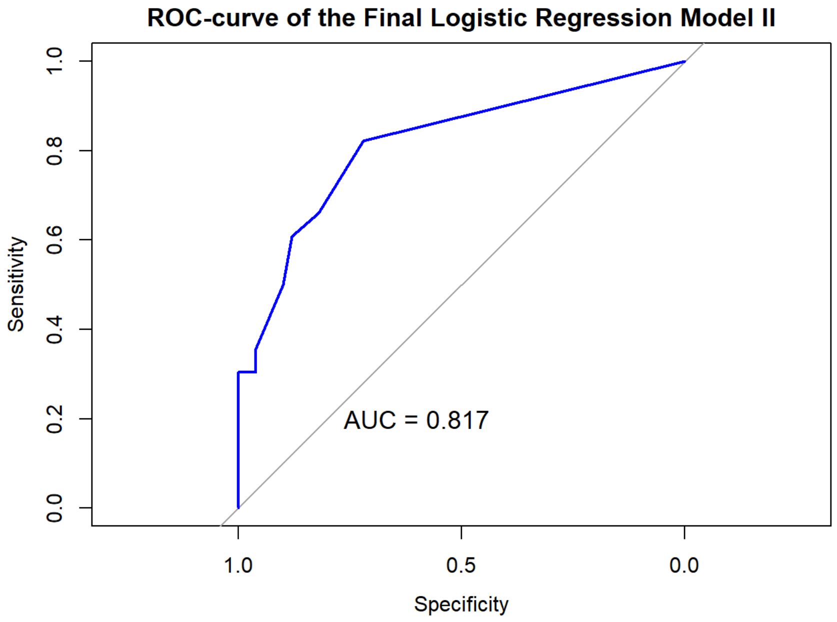

Multivariate Logistic Regression Model III

Includes Predisposition to chronic obstipation (LUTIRE); Severe impact of symptoms on everyday life/activities (ACSS); Multiple uropathogens in urine culture (MIBI); Proteine in urine as “Trace (±)” (Dipstick)

Summary of the Final Logistic Regression Model III

Parameter	Estimate	Std. Error	z value	P-value
(Intercept)	-1.025	0.301	-3.409	0.001
Predisposed to chronic obstipation	1.872	0.732	2.559	0.011
Severe impact of symptoms on everyday life/activities	1.726	0.740	2.334	0.020
Multiple uropathogens	2.495	0.696	3.586	0.000
Proteine in urine as Trace	2.516	1.132	2.222	0.026

ROC Coordinates of the Final Logistic Regression Model III

Measure	Value	CI95

Measure	Value	CI95
threshold	0.47	(0.47–0.76)
sensitivity	0.73	(0.52–0.84)
specificity	0.80	(0.72–0.96)
youden	1.53	(1.38–1.70)
ppv	0.80	(0.74–0.94)
npv	0.73	(0.63–0.82)

Confusion Matrix and Statistics

```
      Reference
```

Prediction 0 1 0 40 15 1 10 41

```
            Accuracy : 0.7642
              95% CI : (0.6718, 0.8412)
 No Information Rate : 0.5283
 P-Value [Acc > NIR] : 4.747e-07

               Kappa : 0.5293
```

Mcnemar’s Test P-Value : 0.4237

```
         Sensitivity : 0.7321
         Specificity : 0.8000
      Pos Pred Value : 0.8039
      Neg Pred Value : 0.7273
          Prevalence : 0.5283
      Detection Rate : 0.3868
```

Detection Prevalence : 0.4811
Balanced Accuracy : 0.7661

```
    'Positive' Class : 1
```

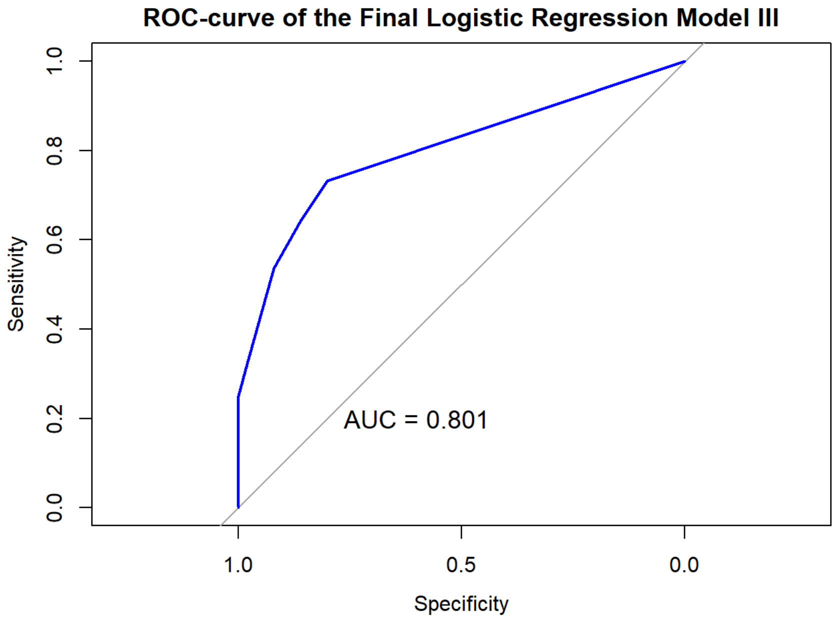

Bootstrapping Model I

Internal validation — Model I (1000 bootstrap resamples)

Apparent_AUC	Optimism_AUC	Corrected_AUC	Apparent_Slope	Optimism_Slope	Corrected_Slope	Cal_Intercept	HL_pvalue	Used_Boot_Iters

Apparent_AUC	Optimism_AUC	Corrected_AUC	Apparent_Slope	Optimism_Slope	Corrected_Slope	Cal_Intercept	HL_pvalue	Used_Boot_Iters
0.838	0.024	0.815	1	0.358	0.642	0	0.301	1000

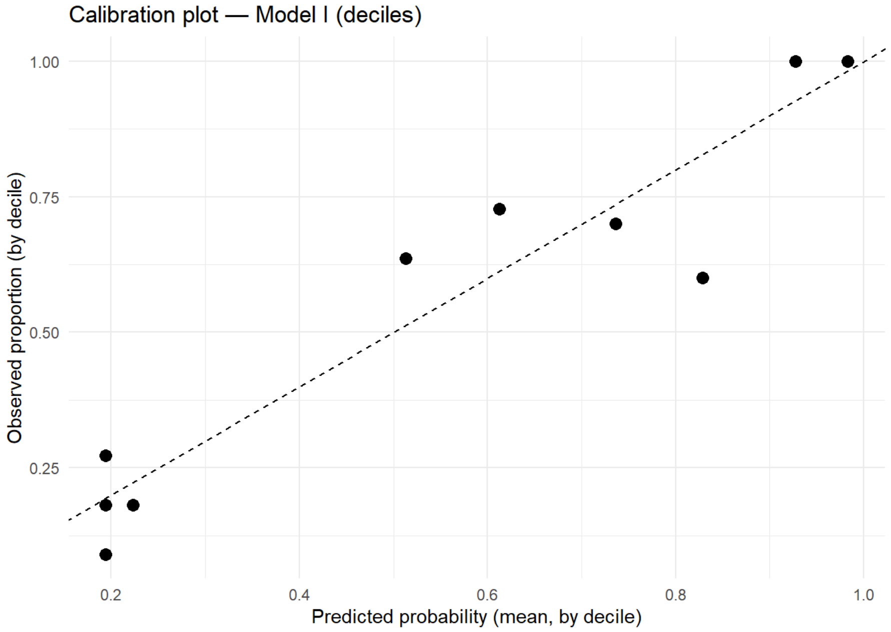

Bootstrapping Model II

Internal validation — Model II (1000 bootstrap resamples)

Apparent_AUC	Optimism_AUC	Corrected_AUC	Apparent_Slope	Optimism_Slope	Corrected_Slope	Cal_Intercept	HL_pvalue	Used_Boot_Iters
0.817	0.017	0.8	1	0.183	0.817	0	0.634	1000

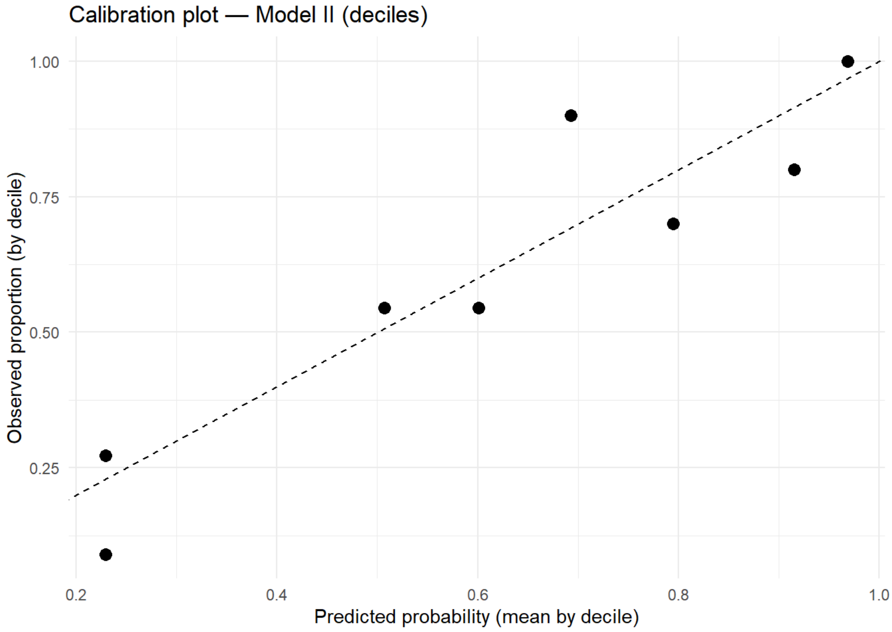

Bootstrapping Model II

Internal validation — Model III (1000 bootstrap resamples)

Apparent_AUC	Optimism_AUC	Corrected_AUC	Apparent_Slope	Optimism_Slope	Corrected_Slope	Cal_Intercept	HL_pvalue	Used_Boot_Iters
0.801	0.016	0.785	1	0.31	0.69	0	0.868	1000

Calibration plot — Model III (deciles)

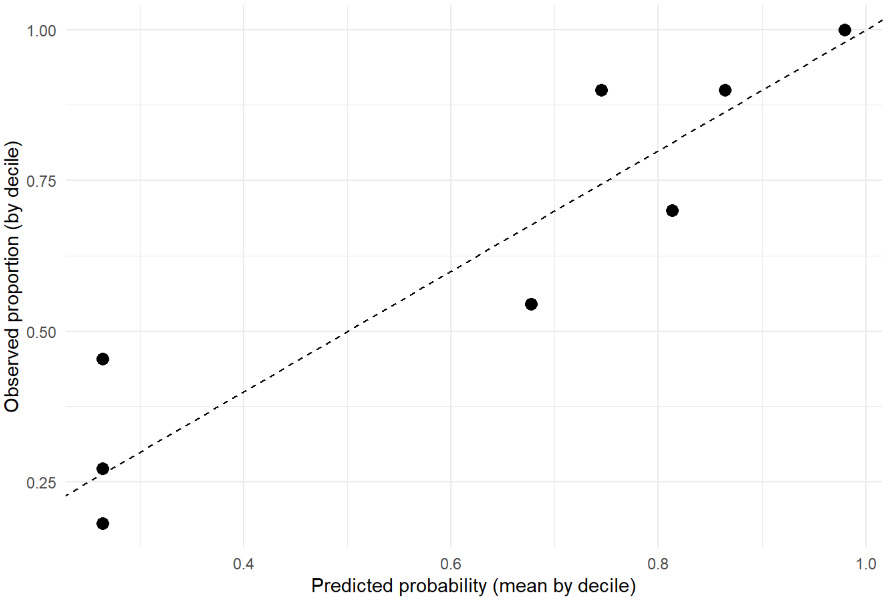

R-Markdown Output of Stepwise Statistical Analysis (Details)

Detailed step-by-step outputs of all statistical analyses and corrections, rendered from the R-Markdown source, are provided below.

Results

Demographics and History (Table 1)

Age statistics (Student/Welch t-test for comparison)

Parameter	Total (n=106)	AC (n=50)	RC (n=56)	P-value (significance)
Age, median (IQR)	36.5 (26.0-58.5)	36.0 (27.2-51.0)	37.5 (24.0-66.5)	0.219 (ns)

Weight statistics (Student/Welch t-test for comparison)

Parameter	Total (n=106)	AC (n=50)	RC (n=56)	P-value (significance)
Weight in kg, median (IQR)	66.0 (57.0-74.8)	63.0 (56.0-70.0)	67.5 (60.0-75.0)	0.191 (ns)

Height statistics (Student/Welch t-test for comparison)

Parameter	Total (n=106)	AC (n=50)	RC (n=56)	P-value (significance)
Height in m, median (IQR)	1.6 (1.6-1.7)	1.6 (1.6-1.7)	1.7 (1.6-1.7)	0.054 (ns)

BMI statistics (Student/Welch t-test for comparison)

Parameter	Total (n=106)	AC (n=50)	RC (n=56)	P-value (significance)
Body-mass index, median (IQR)	23.4 (21.3-27.5)	23.0 (21.3-27.2)	23.4 (21.4-27.7)	0.477 (ns)

Pregnancy Statistics (Fisher’s exact test for comparison of proportions)

Parameter	Total (n=106)	AC (n=50)	RC (n=56)	P-value (significance)
Pregnancy, n (%)	2 (1.9)	1 (2.0)	1 (1.8)	1.000 (ns)

Medical History

Statistics of **previous symptomatic episodes** (Pearson’s chi-squared test for comparison of proportions)

Parameter	Total (n=106)	AC (n=50)	RC (n=56)	P-value
At least one prior symptomatic episode of UTIs in the past 6 months, n (%)	38 (35.8)	7 (14.0)	31 (55.4)	<0.001 (****)
At least one prior symptomatic episode of UTIs in the past 12 months, n (%)	64 (60.4)	8 (16.0)	56 (100.0)	<0.001 (****)

Statistics of **previous symptomatic episodes count** (Student/Welch t-test for comparison)

Parameter	Total (n=106)	AC (n=50)	RC (n=56)	P-value (significance)
Number of prior symptomatic episodes UTIs in the past 6 months, median (IQR)	2.0 (1.0-5.0)	1.0 (0.0-1.0)	3.0 (2.0-5.0)	<0.001 (****)
Number of prior symptomatic episodes UTIs in the past 12 months, median (IQR)	3.0 (0.0-12.0)	0.0 (0.0-0.0)	11.0 (5.0-40.0)	<0.001 (****)

Statistics of **prior antimicrobial treatment** (Comparison using Pearson’s chi-squared proportion test)

Parameter	Total (n=106)	AC (n=50)	RC (n=56)	P-value (significance)
Prior antimicrobial treatment for any reason in the past 3 months, n (%)	41 (38.7)	15 (30.0)	26 (46.4)	0.125 (ns)

Statistics of **prior antimicrobial treatment with single or multiple agents** (Comparison using Pearson’s chi-squared and Fisher’s exact

proportion tests)

Parameter	Total (n=106)	AC (n=50)	RC (n=56)	P-value (significance)
Prior antimicrobial treatment for any reason with a single agent in the past 3 months, n (%)	28 (26.4)	11 (22.0)	17 (30.4)	0.451 (ns)
Prior antimicrobial treatment for any reason with multiple agents in the past 3 months, n (%)	7 (6.6)	1 (2.0)	6 (10.7)	0.117 (ns)

Statistics of **prior prophylactic measures** (Comparison using Pearson's chi-squared and Fisher's exact proportion tests)

Parameter	Total (n=106)	AC (n=50)	RC (n=56)	P-value (significance)
No any prophylactic measure in the past 12 months, n (%)	55 (51.9)	37 (74.0)	18 (32.1)	<0.001 (****)
Single prophylactic measure in the past 12 months, n (%)	8 (7.5)	3 (6.0)	5 (8.9)	0.720 (ns)
Multiple prophylactic measures in the past 12 months, n (%)	43 (40.6)	10 (20.0)	33 (58.9)	<0.001 (***)

Statistics of **duration of a current episode** (Student/Welch t-test for comparison)

Parameter	Total (n=106)	AC (n=50)	RC (n=56)	P-value (significance)
Duration of a current acute episode in days, median (IQR)	3.5 (2.0-6.0)	4.0 (3.0-5.0)	3.0 (2.0-6.5)	0.377 (ns)

Statistics of **current episode attempted to treat** (Comparison using Pearson's chi-squared proportion test)

Parameter	Total (n=106)	AC (n=50)	RC (n=56)	P-value (significance)
Current episode was attempted to treat, n (%)	21 (19.8)	9 (18.0)	12 (21.4)	0.843 (ns)

Risk factors, according to LUTIRE

Statistics of **number of sexual partners within the past year** (Comparison using Pearson's chi-squared and Fisher's exact proportion tests)

Parameter	Total (n=106)	AC (n=50)	RC (n=56)	P-value (significance)
Single sexual partner in the past year, n (%)	92 (86.8)	40 (80.0)	52 (92.9)	0.096 (ns)
Two sexual partners in the past year, n (%)	7 (6.6)	5 (10.0)	2 (3.6)	0.251 (ns)
Three or more sexual partners in the past year, n (%)	7 (6.6)	5 (10.0)	2 (3.6)	0.251 (ns)

Statistics of **bowel function** (Comparison using Pearson's chi-squared and Fisher's exact proportion tests)

Parameter	Total (n=106)	AC (n=50)	RC (n=56)	P-value (significance)
Normal bowel function, n (%)	82 (77.4)	43 (86.0)	39 (69.6)	0.076 (ns)
Predisposed to chronic diarrhea, n (%)	6 (5.7)	4 (8.0)	2 (3.6)	0.418 (ns)
Predisposed to chronic obstipation, n (%)	18 (17.0)	3 (6.0)	15 (26.8)	0.005 (**)

Statistics of **uropathogen, isolated at the preceding episode** (Comparison using Pearson's chi-squared and Fisher's exact proportion tests)

Parameter	Total (n=106)	AC (n=50)	RC (n=56)	P-value (significance)
Known Gram-positive uropathogen isolated at the last acute episode, n (%)	8 (7.5)	2 (4.0)	6 (10.7)	0.277 (ns)
Known Gram-negative uropathogen isolated at the last acute episode, n (%)	25 (23.6)	4 (8.0)	21 (37.5)	<0.001 (***)
No known uropathogen in the past, n (%)	73 (68.9)	44 (88.0)	29 (51.8)	<0.001 (***)

Statistics of **hormonal status** (Comparison using Pearson's chi-squared proportion test)

Parameter	Total (n=106)	AC (n=50)	RC (n=56)	P-value (significance)
Premenopausal hormonal status, n (%)	72 (67.9)	37 (74.0)	35 (62.5)	0.290 (ns)
Postmenopausal hormonal status, n (%)	34 (32.1)	13 (26.0)	21 (37.5)	0.290 (ns)

Statistics of **previous episodes within the last year** (Comparison using Pearson's chi-squared and Fisher's exact proportion tests)

Parameter	Total (n=106)	AC (n=50)	RC (n=56)	P-value (significance)
Up to 2 acute episodes per year, n (%)	52 (49.1)	50 (100.0)	2 (3.6)	<0.001 (****)
Three or more acute episodes per year, n (%)	54 (50.9)	0 (0.0)	54 (96.4)	<0.001 (****)

Statistics of **previous AB therapy due to ASB** (Comparison using Pearson's chi-squared proportion test)

Parameter	Total (n=106)	AC (n=50)	RC (n=56)	P-value (significance)
Any antimicrobial therapy due to ASB in the past, n (%)	28 (26.4)	9 (18.0)	19 (33.9)	0.102 (ns)
No antimicrobial therapy due to ASB in the past, n (%)	78 (73.6)	41 (82.0)	37 (66.1)	0.102 (ns)

Statistics of **porbability of recurence according to LUTIRE** (Student/Welch t-test for comparison)

Parameter	Total (n=106)	AC (n=50)	RC (n=56)	P-value (significance)
Probability of recurrence according to LUTIRE nomogram, median (IQR)	0.30 (0.20-0.40)	0.20 (0.20-0.30)	0.40 (0.30-0.50)	<0.001 (****)

Results of physical examination (Table 2)

Statistics of **ORENUC factors** (Pearson’s chi-squared and Fisher’s exact proportion tests for comparison)

Note: Initial data are manually processed

Parameter	Total (n=106)	AC (n=50)	RC (n=56)	P-value (significance)
O - No known risk factor, n (%)	56 (52.8)	37 (74.0)	19 (33.9)	<0.001 (****)
R - Risk factors for recurrent UTIs, but no risk of more severe outcome, n (%)	32 (30.2)	9 (18.0)	23 (41.1)	0.018 (*)
E - Extra urogenital risk factors with risk of more severe outcome, n (%)	6 (5.7)	0 (0.0)	6 (10.7)	0.028 (*)
N - Nephropathic diseases with risk of more severe outcome, n (%)	1 (0.9)	1 (2.0)	0 (0.0)	0.472 (ns)
U - Urological risk factors with risk of more severe outcome, which can be resolved during therapy, n (%)	8 (7.5)	3 (6.0)	5 (8.9)	0.720 (ns)
C - Permanent urinary catheter and non-resolvable urological risk factors with risk of more severe outcome, n (%)	0 (0.0)	0 (0.0)	0 (0.0)	1.000 (ns)
More than one risk factor, n (%)	3 (2.8)	0 (0.0)	3 (5.4)	0.245 (ns)

Statistics of the **Revealed abnormalities of the urinary tract** (Fisher’s exact proportion test for comparison)

Parameter	Total (n=106)	AC (n=50)	RC (n=56)	P-value (significance)
Revealed abnormalities of the urinary tract, n (%)	9 (8.5)	4 (8.0)	5 (8.9)	1.000 (ns)

Statistics of the **additional conditions** according to the ACSS (Fisher’s exact proportion test for comparison)

Parameter	Total (n=106)	AC (n=50)	RC (n=56)	P-value (significance)
Menstruations, n (%)	12 (11.3)	7 (14.0)	5 (8.9)	0.542 (ns)

Parameter	Total (n=106)	AC (n=50)	RC (n=56)	P-value (significance)
Premenstrual symptoms, n (%)	9 (8.5)	6 (12.0)	3 (5.4)	0.301 (ns)
Symptoms of menopause, n (%)	9 (8.5)	8 (16.0)	1 (1.8)	0.012 (*)
Pregnancy, n (%)	2 (1.9)	1 (2.0)	1 (1.8)	1.000 (ns)
Sugar diabetes, n (%)	9 (8.5)	4 (8.0)	5 (8.9)	1.000 (ns)

Self-Reporting data according the ACSS and EQ-5D-3L (Table 3)

Statistics of the **Symptom Severity** according to the ACSS (Pearson's chi-squared and Fisher's exact proportion tests for comparison)

Parameter	Total (n=106)	AC (n=50)	RC (n=56)	P-value (significance)
**Frequent urination, n (%)**	96 (90.6)	46 (92.0)	50 (89.3)	0.885 (ns)
Mild, n (%)	21 (19.8)	11 (22.0)	10 (17.9)	0.772 (ns)
Moderate, n (%)	40 (37.7)	19 (38.0)	21 (37.5)	1.000 (ns)
Severe, n (%)	35 (33.0)	16 (32.0)	19 (33.9)	0.997 (ns)
**Urgent urination, n (%)**	96 (90.6)	45 (90.0)	51 (91.1)	1.000 (ns)
Mild, n (%)	18 (17.0)	10 (20.0)	8 (14.3)	0.601 (ns)
Moderate, n (%)	39 (36.8)	18 (36.0)	21 (37.5)	1.000 (ns)
Severe, n (%)	39 (36.8)	17 (34.0)	22 (39.3)	0.718 (ns)
**Painful urination, n (%)**	92 (86.8)	44 (88.0)	48 (85.7)	0.952 (ns)

Parameter	Total (n=106)	AC (n=50)	RC (n=56)	P-value (significance)
Mild, n (%)	15 (14.2)	7 (14.0)	8 (14.3)	1.000 (ns)
Moderate, n (%)	32 (30.2)	19 (38.0)	13 (23.2)	0.149 (ns)
Severe, n (%)	45 (42.5)	18 (36.0)	27 (48.2)	0.283 (ns)
**Sense of incomplete bladder emptying, n (%)**	82 (77.4)	36 (72.0)	46 (82.1)	0.311 (ns)
Mild, n (%)	20 (18.9)	5 (10.0)	15 (26.8)	0.050 (ns)
Moderate, n (%)	29 (27.4)	19 (38.0)	10 (17.9)	0.035 (*)
Severe, n (%)	33 (31.1)	12 (24.0)	21 (37.5)	0.198 (ns)
**Suprapubic pain, n (%)**	84 (79.2)	39 (78.0)	45 (80.4)	0.953 (ns)
Mild, n (%)	16 (15.1)	8 (16.0)	8 (14.3)	1.000 (ns)
Moderate, n (%)	28 (26.4)	14 (28.0)	14 (25.0)	0.897 (ns)
Severe, n (%)	40 (37.7)	17 (34.0)	23 (41.1)	0.583 (ns)
**Visible blood in urine, n (%)**	29 (27.4)	17 (34.0)	12 (21.4)	0.218 (ns)
Mild, n (%)	12 (11.3)	6 (12.0)	6 (10.7)	1.000 (ns)
Moderate, n (%)	11 (10.4)	8 (16.0)	3 (5.4)	0.110 (ns)
Severe, n (%)	6 (5.7)	3 (6.0)	3 (5.4)	1.000 (ns)
**Flank pain, n (%)**	50 (47.2)	27 (54.0)	23 (41.1)	0.256 (ns)
Mild, n (%)	22 (20.8)	9 (18.0)	13 (23.2)	0.674 (ns)
Moderate, n (%)	15 (14.2)	8 (16.0)	7 (12.5)	0.813 (ns)

Parameter	Total (n=106)	AC (n=50)	RC (n=56)	P-value (significance)
Severe, n (%)	13 (12.3)	10 (20.0)	3 (5.4)	0.035 (*)
**Abnormal vaginal discharge, n (%)**	29 (27.4)	16 (32.0)	13 (23.2)	0.427 (ns)
Mild, n (%)	19 (17.9)	12 (24.0)	7 (12.5)	0.198 (ns)
Moderate, n (%)	4 (3.8)	1 (2.0)	3 (5.4)	0.620 (ns)
Severe, n (%)	6 (5.7)	3 (6.0)	3 (5.4)	1.000 (ns)
**Urethral discharge, n (%)**	4 (3.8)	3 (6.0)	1 (1.8)	0.341 (ns)
Mild, n (%)	1 (0.9)	1 (2.0)	0 (0.0)	0.472 (ns)
Moderate, n (%)	2 (1.9)	2 (4.0)	0 (0.0)	0.220 (ns)
Severe, n (%)	1 (0.9)	0 (0.0)	1 (1.8)	1.000 (ns)
**Sense of fever/chills, n (%)**	21 (19.8)	11 (22.0)	10 (17.9)	0.772 (ns)
Mild, n (%)	10 (9.4)	5 (10.0)	5 (8.9)	1.000 (ns)
Moderate, n (%)	6 (5.7)	3 (6.0)	3 (5.4)	1.000 (ns)
Severe, n (%)	5 (4.7)	3 (6.0)	2 (3.6)	0.665 (ns)
**General discomfort, n (%)**	104 (98.1)	49 (98.0)	55 (98.2)	1.000 (ns)
Mild, n (%)	23 (21.7)	13 (26.0)	10 (17.9)	0.436 (ns)
Moderate, n (%)	60 (56.6)	28 (56.0)	32 (57.1)	1.000 (ns)
Severe, n (%)	21 (19.8)	8 (16.0)	13 (23.2)	0.493 (ns)
**Impact on everyday life/activities, n (%)**	99 (93.4)	46 (92.0)	53 (94.6)	0.877 (ns)
Mild, n (%)	40 (37.7)	20 (40.0)	20 (35.7)	0.800 (ns)

Parameter	Total (n=106)	AC (n=50)	RC (n=56)	P-value (significance)
Moderate, n (%)	41 (38.7)	23 (46.0)	18 (32.1)	0.207 (ns)
Severe, n (%)	18 (17.0)	3 (6.0)	15 (26.8)	0.005 (**)
**Impact on social life/activities, n (%)**	93 (87.7)	43 (86.0)	50 (89.3)	0.827 (ns)
Mild, n (%)	34 (32.1)	18 (36.0)	16 (28.6)	0.542 (ns)
Moderate, n (%)	39 (36.8)	18 (36.0)	21 (37.5)	1.000 (ns)
Severe, n (%)	20 (18.9)	7 (14.0)	13 (23.2)	0.336 (ns)

Statistics of the **general health condition** according to the EQ-5D-3L (Pearson's chi-squared and Fisher's exact proportion tests and Student/Welch T-test for comparison)

Item	Parameter	Total (n=106)	AC (n=50)	RC (n=56)	P-value (significance)
**Mobility**	No problems in walking about, (n%)	60 (56.6)	33 (66.0)	27 (48.2)	0.099 (ns)
**Mobility**	Some problems in walking about, (n%)	33 (31.1)	13 (26.0)	20 (35.7)	0.385 (ns)
**Mobility**	Confined to bed, (n%)	3 (2.8)	0 (0.0)	3 (5.4)	0.245 (ns)
**Mobility**	Missing values, (n%)	10 (9.4)	4 (8.0)	6 (10.7)	0.746 (ns)
**Self-care**	No problems with self-care, (n%)	86 (81.1)	42 (84.0)	44 (78.6)	0.642 (ns)
**Self-care**	Some problems washing or dressing, (n%)	10 (9.4)	4 (8.0)	6 (10.7)	0.746 (ns)
**Self-care**	Unable to wash or dress, (n%)	0 (0.0)	0 (0.0)	0 (0.0)	1.000 (ns)
**Self-care**	Missing values, (n%)	10 (9.4)	4 (8.0)	6 (10.7)	0.746 (ns)

Item	Parameter	Total (n=106)	AC (n=50)	RC (n=56)	P-value (significance)
**Usual activities**	No problems with performing usual activities, (n%)	59 (55.7)	35 (70.0)	24 (42.9)	0.009 (**)
**Usual activities**	Some problems with performing usual activities, (n%)	30 (28.3)	10 (20.0)	20 (35.7)	0.115 (ns)
**Usual activities**	Unable to perform usual activities, (n%)	7 (6.6)	1 (2.0)	6 (10.7)	0.117 (ns)
**Usual activities**	Missing values, (n%)	10 (9.4)	4 (8.0)	6 (10.7)	0.746 (ns)
**Pain or discomfort**	No pain or discomfort, (n%)	27 (25.5)	14 (28.0)	13 (23.2)	0.733 (ns)
**Pain or discomfort**	Moderate pain or discomfort, (n%)	51 (48.1)	23 (46.0)	28 (50.0)	0.828 (ns)
**Pain or discomfort**	Extreme pain or discomfort, (n%)	18 (17.0)	9 (18.0)	9 (16.1)	0.996 (ns)
**Pain or discomfort**	Missing values, (n%)	10 (9.4)	4 (8.0)	6 (10.7)	0.746 (ns)
**Anxiety/depression**	Not anxious or depressed, (n%)	62 (58.5)	33 (66.0)	29 (51.8)	0.199 (ns)
**Anxiety/depression**	Moderately anxious or depressed, (n%)	27 (25.5)	13 (26.0)	14 (25.0)	1.000 (ns)
**Anxiety/depression**	Extremely anxious or depressed, (n%)	7 (6.6)	0 (0.0)	7 (12.5)	0.014 (*)
**Anxiety/depression**	Missing values, (n%)	10 (9.4)	4 (8.0)	6 (10.7)	0.746 (ns)
**General health status**	General health status, median (IQR)	60.0 (30.0-80.0)	60.0 (30.0-81.2)	60.0 (32.0-80.0)	0.820 (ns)

Self-Reporting Symptom Scores according to the ACSS (Suppl. Table 3)

Statistics of the **Symptom Scores** according to the ACSS (Wilcoxon-Mann-Whitney and Student/Welch t-test for comparison with Benjamini-Hochberg adjustment for multiple comparisons to control the

false discovery rate)

Parameter	Total (n=106)	AC (n=50)	RC (n=56)	P-value (significance)
Frequent urination, median (IQR)	2.0 (1.0-3.0)	2.0 (1.0-3.0)	2.0 (1.0-3.0)	0.899 (ns)
Urgent urination, median (IQR)	2.0 (1.0-3.0)	2.0 (1.0-3.0)	2.0 (2.0-3.0)	0.634 (ns)
Painful urination, median (IQR)	2.0 (1.0-3.0)	2.0 (1.2-3.0)	2.0 (1.0-3.0)	0.634 (ns)
Sense of incomplete bladder emptying, median (IQR)	2.0 (1.0-3.0)	2.0 (0.0-2.0)	2.0 (1.0-3.0)	0.634 (ns)
Suprapubic pain, median (IQR)	2.0 (1.0-3.0)	2.0 (1.0-3.0)	2.0 (1.0-3.0)	0.634 (ns)
Visible blood in urine, median (IQR)	0.0 (0.0-1.0)	0.0 (0.0-1.0)	0.0 (0.0-0.0)	0.445 (ns)
**Summary score of the ‘Typical’ domain, median (IQR)**	9.5 (8.0-12.0)	9.0 (8.0-12.0)	10.0 (8.0-12.2)	0.730 (ns)
Flank pain, median (IQR)	0.0 (0.0-2.0)	1.0 (0.0-2.0)	0.0 (0.0-1.0)	0.445 (ns)
Abnormal vaginal discharge, median (IQR)	0.0 (0.0-1.0)	0.0 (0.0-1.0)	0.0 (0.0-0.0)	0.634 (ns)
Urethral discharge, median (IQR)	0.0 (0.0-0.0)	0.0 (0.0-0.0)	0.0 (0.0-0.0)	0.550 (ns)
Sense of fever/chills, median (IQR)	0.0 (0.0-0.0)	0.0 (0.0-0.0)	0.0 (0.0-0.0)	0.658 (ns)
**Summary score of the ‘Differential’ domain, median (IQR)**	1.0 (0.0-3.0)	2.0 (0.0-3.0)	1.0 (0.0-2.0)	0.445 (ns)
General discomfort, median (IQR)	2.0 (2.0-2.0)	2.0 (1.0-2.0)	2.0 (2.0-2.0)	0.530 (ns)
Impact on everyday life/activities, median (IQR)	2.0 (1.0-2.0)	2.0 (1.0-2.0)	2.0 (1.0-3.0)	0.445 (ns)
Impact on social life/activities, median (IQR)	2.0 (1.0-2.0)	1.5 (1.0-2.0)	2.0 (1.0-2.0)	0.520 (ns)

Parameter	Total (n=106)	AC (n=50)	RC (n=56)	P-value (significance)
**Summary score of the ‘QoL’ domain, median (IQR)**	5.0 (4.0-7.0)	5.0 (3.0-6.0)	5.5 (4.0-7.2)	0.445 (ns)

Results of lab and microbiological analysis of urine (Table 4)

Statistics of **Urine leucocyte-esterase test** (Pearson’s chi-squared and Fisher’s exact proportion tests for comparison)

Result	Total (n=106)	AC (n=50)	RC (n=56)	P-value (significance)
Negative LEU, n (%)	12 (11.3)	2 (4.0)	10 (17.9)	0.032 (*)
Trace LEU, n (%)	5 (4.7)	1 (2.0)	4 (7.1)	0.367 (ns)
Small (1+) LEU, n (%)	12 (11.3)	4 (8.0)	8 (14.3)	0.369 (ns)
Moderate (2+) LEU, n (%)	28 (26.4)	18 (36.0)	10 (17.9)	0.001 (**)
Large (3+) LEU, n (%)	49 (46.2)	25 (50.0)	24 (42.9)	1.000 (ns)

Note: Leucocyturia as Negative - 0 WBC/μl; Trace (±) - up to 15 WBC/μl; Small (1+) - 16–75 WBC/μl; Moderate (2+) - 76–125 WBC/μl; Large (3+) - 126 RBC/μl and above

Statistics of **Urine nitrite test** (Pearson’s chi-squared proportion test for comparison)

Parameter	Total (n=106)	AC (n=50)	RC (n=56)	P-value (significance)
Positive nitrite test, n (%)	44 (41.5)	20 (40.0)	24 (42.9)	0.920 (ns)

Statistics of **Urine nitrite AND leucocyte-esterase tests** (Pearson’s chi-squared and Fisher exact proportion tests for comparison)

Parameter	Total (n=106)	AC (n=50)	RC (n=56)	P-value (significance)
Both nitrite and lecocyte-esterase tests are negative, n (%)	8 (7.5)	1 (2.0)	7 (12.5)	0.063 (ns)

Parameter	Total (n=106)	AC (n=50)	RC (n=56)	P-value (significance)
Both nitrite and lecocyte-esterase tests are positive, n (%)	36 (34.0)	18 (36.0)	18 (32.1)	0.831 (ns)

Note: Negative pyuria in this case includes leucocyte-esterase results as Negative (0 WBC/μl) and Trace (up to 15 WBC/μl)

Statistics of **Urine RBC test** (Pearson's chi-squared and Fisher exact proportion tests for comparison)

Parameter	Total (n=106)	AC (n=50)	RC (n=56)	P-value (significance)
RBC in urine as Negative, n(%)	79 (74.5)	40 (80.0)	39 (69.6)	0.318 (ns)
RBC in urine as Trace (±), n(%)	1 (0.9)	1 (2.0)	0 (0.0)	0.472 (ns)
RBC in urine as Small (1+), n(%)	1 (0.9)	1 (2.0)	0 (0.0)	0.472 (ns)
RBC in urine as Moderate (2+), n(%)	8 (7.5)	0 (0.0)	8 (14.3)	0.006 (**)
RBC in urine as Large (3+), n(%)	17 (16.0)	8 (16.0)	9 (16.1)	1.000 (ns)

Note: Haematuria as Negative - 0 RBC/μl; Trace (±) - up to 5 RBC/μl; Small (1+) - 6–10 RBC/μl; Moderate (2+) - 11–25 RBC/μl; Large (3+) - 26-50 RBC/μl and above

Statistics of **Urine proteine test** (Pearson's chi-squared and Fisher exact proportion tests for comparison)

Parameter	Total (n=106)	AC (n=50)	RC (n=56)	P-value (significance)
Proteine in urine as Negative, n(%)	89 (84.0)	44 (88.0)	45 (80.4)	0.421 (ns)
Proteine in urine as Trace (±), n(%)	10 (9.4)	1 (2.0)	9 (16.1)	0.018 (*)
Proteine in urine as Small (1+), n(%)	2 (1.9)	1 (2.0)	1 (1.8)	1.000 (ns)
Proteine in urine as Moderate (2+), n(%)	3 (2.8)	2 (4.0)	1 (1.8)	0.601 (ns)
Proteine in urine as Large (3+), n(%)	2 (1.9)	2 (4.0)	0 (0.0)	0.220 (ns)

Note: Proteinuria as Negative - 0 mg/dL; Trace (±) - up to 30 mg/dL; Small (1+) - 31–50 mg/dL; Moderate (2+) - 51–100 mg/dL; Large (3+) - 101 mg/dL and above

Statistics of **Positive urine culture** (Pearson's chi-squared proportion test for comparison)

Parameter	Total (n=106)	AC (n=50)	RC (n=56)	P-value (significance)
Positive urine culture (≥ 10^3), n (%)	87 (82.1)	33 (66.0)	54 (96.4)	<0.001 (***)

Statistics of **Positive pyuria and urine culture** (Pearson's chi-squared proportion test for comparison)

Parameter	Total (n=106)	AC (n=50)	RC (n=56)	P-value (significance)
Positive urine culture AND positive pyuria, n (%)	71 (67.0)	30 (60.0)	41 (73.2)	0.216 (ns)

Note: Positive pyuria in this case includes leucocyte-esterase results as Small (+1), Moderate (2+) and Large (3+); Positive urine culture when CFU of first or second pathogen ≥10^3

Statistics of **Number of isolated uropathogens in urine culture** (Pearson's chi-squared and Fisher exact proportion tests for comparison)

Parameter	Total (n=106)	AC (n=50)	RC (n=56)	P-value (significance)
Single uropathogen, n (%)	63 (59.4)	30 (60.0)	33 (58.9)	1.000 (ns)
Multiple uropathogens, n (%)	24 (22.6)	3 (6.0)	21 (37.5)	<0.001 (***)

Statistics of the **First uropathogen in urine culture (by Gram staining)** (Pearson's chi-squared and Fisher exact proportion tests for comparison)

Parameter	Total (n=87)	AC (n=33)	RC (n=54)	P-value (significance)
Gram positive species, n (%)	15 (17.2)	3 (9.1)	12 (22.2)	0.306 (ns)
Gram negative species, n (%)	65 (74.7)	25 (75.8)	40 (74.1)	0.985 (ns)
Other/Unknown, n (%)	7 (8.0)	5 (15.2)	2 (3.7)	0.174 (ns)

Statistics of the **First uropathogen in urine culture (by species)** (Pearson's chi-squared and Fisher exact proportion tests for comparison)

Gram staining	Parameter	Total (n=87)	AC (n=33)	RC (n=54)	P-value (significance)
Gram positive species	Enterococcus sp., n (%)	4 (4.6)	0 (0.0)	4 (7.4)	0.293 (ns)
Gram positive species	G. vaginalis, n (%)	1 (1.1)	0 (0.0)	1 (1.9)	1.000 (ns)
Gram positive species	Lactobacillus sp., n (%)	1 (1.1)	0 (0.0)	1 (1.9)	1.000 (ns)
Gram positive species	Other Gram+ bacteria, n (%)	1 (1.1)	0 (0.0)	1 (1.9)	1.000 (ns)
Gram positive species	Other coagulase-negative Staphylococci, n (%)	3 (3.4)	0 (0.0)	3 (5.6)	0.285 (ns)
Gram positive species	Staphylococcus aureus, n (%)	1 (1.1)	1 (3.0)	0 (0.0)	0.379 (ns)
Gram positive species	Staphylococcus saprophyticus, n (%)	4 (4.6)	2 (6.1)	2 (3.7)	0.632 (ns)
Gram negative species	E. coli, n (%)	55 (63.2)	22 (66.7)	33 (61.1)	0.770 (ns)
Gram negative species	Klebsiella sp., n (%)	8 (9.2)	3 (9.1)	5 (9.3)	1.000 (ns)
Gram negative species	Pseudomonas aeruginosa, n (%)	2 (2.3)	0 (0.0)	2 (3.7)	0.524 (ns)
Other/Unknown	Mixed flora, n (%)	7 (8.0)	5 (15.2)	2 (3.7)	0.099 (ns)

Statistics of the **CFU of the first pathogen** (Pearson's chi-squared and Fisher exact proportion tests for comparison)

Parameter	Total (n=87)	AC (n=33)	RC (n=54)	P-value (significance)
CFU>=10^3, n (%)	6 (6.9)	4 (12.1)	2 (3.7)	0.195 (ns)

Parameter	Total (n=87)	AC (n=33)	RC (n=54)	P-value (significance)
CFU>=10^4, n (%)	27 (31.0)	12 (36.4)	15 (27.8)	0.548 (ns)
CFU>=10^5, n (%)	20 (23.0)	6 (18.2)	14 (25.9)	0.568 (ns)
CFU>=10^6, n (%)	34 (39.1)	11 (33.3)	23 (42.6)	0.527 (ns)

Statistics of the **General non-susceptibility of the first pathogen** (Pearson's chi-squared proportion test for comparison)

Parameter	Total (n=808)	AC (n=284)	RC (n=524)	P-value (significance)
General non-susceptibility rate of all first uropathogens, to all tested classes of antimicrobial agents, n (%)	140 (17.3)	61 (21.5)	79 (15.1)	0.028 (*)

Statistics of the **Second uropathogen in urine culture (by Gram staining)** (Fisher exact proportion test for comparison)

Parameter	Total (n=24)	AC (n=3)	RC (n=21)	P-value (significance)
Gram positive species, n (%)	15 (62.5)	1 (33.3)	14 (66.7)	0.533 (ns)
Gram negative species, n (%)	7 (29.2)	1 (33.3)	6 (28.6)	1.000 (ns)
Mixed flora (unknown Gram stain), n (%)	2 (8.3)	1 (33.3)	1 (4.8)	0.239 (ns)

Statistics of the **Second uropathogen in urine culture (by species)** (Fisher exact proportion test for comparison)

Gram staining	Parameter	Total (n=24)	AC (n=3)	RC (n=21)	P-value (significance)
Gram positive species	Aerococcus urinae, n (%)	2 (8.3)	0 (0.0)	2 (9.5)	1.000 (ns)
Gram positive species	Enterococcus sp., n (%)	4 (16.7)	0 (0.0)	4 (19.0)	1.000 (ns)
Gram positive species	Group B beta-haemolytic Streptococci, n (%)	4 (16.7)	1 (33.3)	3 (14.3)	0.437 (ns)
Gram positive species	Lactobacillus sp., n (%)	1 (4.2)	0 (0.0)	1 (4.8)	1.000 (ns)

Gram staining	Parameter	Total (n=24)	AC (n=3)	RC (n=21)	P-value (significance)
Gram positive species	Mixed flora (Gram positive), n (%)	3 (12.5)	0 (0.0)	3 (14.3)	1.000 (ns)
Gram positive species	Staphylococcus epidermidis, n (%)	1 (4.2)	0 (0.0)	1 (4.8)	1.000 (ns)
Gram negative species	Citrobacter sp., n (%)	1 (4.2)	0 (0.0)	1 (4.8)	1.000 (ns)
Gram negative species	Enterobacter sp., n (%)	1 (4.2)	0 (0.0)	1 (4.8)	1.000 (ns)
Gram negative species	Klebsiella sp., n (%)	1 (4.2)	0 (0.0)	1 (4.8)	1.000 (ns)
Gram negative species	Morganella sp., n (%)	1 (4.2)	0 (0.0)	1 (4.8)	1.000 (ns)
Gram negative species	Other Enterobacteriaceae, n (%)	1 (4.2)	1 (33.3)	0 (0.0)	0.125 (ns)
Gram negative species	Proteus sp., n (%)	2 (8.3)	0 (0.0)	2 (9.5)	1.000 (ns)
Other/Unknown	Mixed flora (unknown Gram stain), n (%)	2 (8.3)	1 (33.3)	1 (4.8)	0.239 (ns)

Statistics of the **CFU of the second pathogen** (Fisher exact proportion test for comparison)

Parameter	Total (n=24)	AC (n=3)	RC (n=21)	P-value (significance)
CFU>=10^3, n (%)	7 (29.2)	2 (66.7)	5 (23.8)	0.267 (ns)
CFU>=10^4, n (%)	6 (25.0)	1 (33.3)	5 (23.8)	1.000 (ns)
CFU>=10^5, n (%)	7 (29.2)	0 (0.0)	7 (33.3)	0.693 (ns)
CFU>=10^6, n (%)	4 (16.7)	0 (0.0)	4 (19.0)	1.000 (ns)

Statistics of the **General non-susceptibility of the second**

pathogen (Fisher's exact proportion test for comparison)

Parameter	Total (n=136)	AC (n=1)	RC (n=135)	P-value (significance)
General non-susceptibility rate of all second uropathogens, to all tested classes of antimicrobial agents, n (%)	31 (22.8)	0 (0.0)	31 (23.0)	1.000 (ns)

Statistics of the **Non-susceptibility of E. coli to different classes of antimicrobials** (Pearson's chi-squared and Fisher's exact proportion tests for comparison)

Parameter	Total (n=97)	AC (n=51)	RC (n=46)	P-value (significance)
Aminoglycosides, n (%)	1 (1.0)	1 (2.0)	0 (0.0)	1.000 (ns)
1-st gen. Cephalosporins, n (%)	3 (3.1)	3 (5.9)	0 (0.0)	0.244 (ns)
2-nd gen. Cephalosporins, n (%)	28 (28.9)	9 (17.6)	19 (41.3)	0.019 (*)
3-rd gen. Cephalosporins, n (%)	5 (5.2)	5 (9.8)	0 (0.0)	0.058 (ns)
5-th gen. Cephalosporins, n (%)	1 (1.0)	1 (2.0)	0 (0.0)	1.000 (ns)
Fosfomycin, n (%)	1 (1.0)	1 (2.0)	0 (0.0)	1.000 (ns)
Fluoroquinolones, n (%)	12 (12.4)	8 (15.7)	4 (8.7)	0.364 (ns)
Penicillins, n (%)	22 (22.7)	10 (19.6)	12 (26.1)	0.604 (ns)
Penicillins + β-lactamase inhibitors, n (%)	14 (14.4)	7 (13.7)	7 (15.2)	1.000 (ns)
Tetracyclines, n (%)	2 (2.1)	2 (3.9)	0 (0.0)	0.496 (ns)
Trimethoprim, n (%)	8 (8.2)	4 (7.8)	4 (8.7)	1.000 (ns)

Relative risk calculation based on proportions. Parameter as an Outcome, RC as an Exposure" (Table 5)

Chronic obstipation

```
         Outcome +    Outcome -      Total                   Inc risk *
```

Exposed + 15 3 18 83.33 (58.58 to 96.42) Exposed - 41 47 88 46.59 (35.88 to 57.54) Total 56 50 106 52.83 (42.89 to 62.60)

Point estimates and 95% CIs:

Inc risk ratio 1.79 (1.32, 2.43) Inc odds ratio 5.73 (1.55, 21.21) Attrib risk in the exposed * 36.74 (16.62, 56.87) Attrib fraction in the exposed (%) 44.09 (24.19, 58.77) Attrib risk in the population * 6.24 (-7.87, 20.34) Attrib fraction in the population (%) 11.81 (2.97, 19.84) ——————————————————————- Uncorrected chi2 test that OR = 1: chi2(1) = 8.095 Pr>chi2 = 0.004 Fisher exact test that OR = 1: Pr>chi2 = 0.005 Wald confidence limits CI: confidence interval * Outcomes per 100 population units

Measures of association strength: The outcome incidence risk among the exposed was 1.79 (95% CI 1.32 to 2.43) times the outcome incidence risk among the unexposed: exposure increased the outcome incidence risk among the exposed.

The outcome incidence odds among the exposed was 5.73 (95% CI 1.55 to 21.21) times the outcome incidence odds among the unexposed: exposure increased the outcome incidence odds among the exposed.

Measures of effect in the exposed: Exposure changed the outcome incidence risk in the exposed by 36.74 (95% CI 16.62 to 56.87) per 100 population units. 44.1% of outcomes in the exposed were attributable to exposure (95% CI 24.2% to 58.8%).

Number needed to treat for benefit (NNTB) and harm (NNTH): Exposure increased the outcome incidence risk in the exposed. The number needed to treat (expose) to increase the outcome frequency by one was 3 (95% CI 2 to 6).

Measures of effect in the population: Exposure changed the outcome incidence risk in the population by 6.24 (95% CI -7.87 to 20.34) per 100 population units. 11.8% of outcomes in the population were attributable to exposure (95% CI 3% to 19.8%).

E - Extra urogenital risk factors

```
          Outcome +    Outcome -      Total                 Inc risk *
```

Exposed + 6 0 6 100.00 (54.07 to 100.00) Exposed - 50 50 100 50.00 (39.83 to 60.17) Total 56 50 106 52.83 (42.89 to 62.60)

Point estimates and 95% CIs:

Inc risk ratio 2.00 (1.64, 2.43) Inc odds ratio NaN (NaN, NaN) Attrib risk in the exposed * 50.00 (40.20, 59.80) Attrib fraction in the exposed (%) 50.00 (39.17, 58.90) Attrib risk in the population * 2.83 (-10.82, 16.48) Attrib fraction in the population (%) 5.36 (0.84, 9.67) ——————————————————————- Yates corrected chi2 test that OR = 1: chi2(1) = 3.849 Pr>chi2 = 0.050 Fisher exact test that OR = 1: Pr>chi2 = 0.028 Wald confidence limits CI: confidence interval * Outcomes per 100 population units

Measures of association strength: The outcome incidence risk among the exposed was 2 (95% CI 1.64 to 2.43) times the outcome incidence risk among the unexposed: exposure increased the outcome incidence risk among the exposed.

The outcome incidence odds among the exposed was NaN (95% CI NaN to NaN) times the outcome incidence odds among the unexposed: exposure NA the outcome incidence odds among the exposed.

Measures of effect in the exposed: Exposure changed the outcome incidence risk in the exposed by 50 (95% CI 40.2 to 59.8) per 100 population units. 50% of outcomes in the exposed were attributable to exposure (95% CI 39.2% to 58.9%).

Number needed to treat for benefit (NNTB) and harm (NNTH): Exposure increased the outcome incidence risk in the exposed. The number needed to treat (expose) to increase the outcome frequency by one was 2 (95% CI 2 to 2).

Measures of effect in the population: Exposure changed the outcome incidence risk in the population by 2.83 (95% CI -10.82 to 16.48) per 100 population units. 5.4% of outcomes in the population were attributable to exposure (95% CI 0.8% to 9.7%).

Moderate sense of incomplete bladder emptying

```
         Outcome +    Outcome -      Total                Inc risk *
```

Exposed + 10 19 29 34.48 (17.94 to 54.33) Exposed - 46 31 77 59.74 (47.94 to 70.77) Total 56 50 106 52.83 (42.89 to 62.60)

Point estimates and 95% CIs:

Inc risk ratio 0.58 (0.34, 0.98) Inc odds ratio 0.35 (0.15, 0.86) Attrib risk in the exposed * -25.26 (-45.73, -4.78) Attrib fraction in the exposed (%) -73.25 (-195.55, -1.55) Attrib risk in the population * -6.91 (-21.41, 7.59) Attrib fraction in the population (%) -13.08 (-25.43, -1.95) ——————————————————————- Uncorrected chi2 test that OR = 1: chi2(1) = 5.393 Pr>chi2 = 0.020 Fisher exact test that OR = 1: Pr>chi2 = 0.029 Wald confidence limits CI: confidence interval * Outcomes per 100 population units

Measures of association strength: The outcome incidence risk among the exposed was 0.58 (95% CI 0.34 to 0.98) times the outcome incidence risk among the unexposed: exposure decreased the outcome incidence risk among the exposed.

The outcome incidence odds among the exposed was 0.35 (95% CI 0.15 to 0.86) times the outcome incidence odds among the unexposed: exposure decreased the outcome incidence odds among the exposed.

Measures of effect in the exposed: Exposure changed the outcome incidence risk in the exposed by -25.26 (95% CI -45.73 to -4.78) per 100 population units. -73.2% of outcomes in the exposed were attributable to exposure (95% CI -195.6% to -1.6%).

Number needed to treat for benefit (NNTB) and harm (NNTH): Exposure decreased the outcome incidence risk in the exposed. The number needed to treat (expose) to decrease the outcome frequency by one was 4 (95% CI 21 to 2).

Measures of effect in the population: Exposure changed the outcome incidence risk in the population by -6.91 (95% CI -21.41 to 7.59) per 100 population units. -13.1% of outcomes in the population were attributable to exposure (95% CI -25.4% to -1.9%).

Severe flank pain

```
         Outcome +    Outcome -      Total                Inc risk *
```

Exposed + 3 10 13 23.08 (5.04 to 53.81) Exposed - 53 40 93 56.99 (46.31 to 67.22) Total 56 50 106 52.83 (42.89 to 62.60)

Point estimates and 95% CIs:

Inc risk ratio 0.40 (0.15, 1.11) Inc odds ratio 0.23 (0.06, 0.88) Attrib risk in the exposed * -33.91 (-58.93, -8.90) Attrib fraction in the exposed (%) -146.95 (-576.71, 9.88) Attrib risk in the population * -4.16 (-18.00, 9.68) Attrib fraction in the population (%) -7.87 (-15.38, -0.86) ——————————————————————- Uncorrected chi2 test that OR = 1: chi2(1) = 5.264 Pr>chi2 = 0.022 Fisher exact test that OR = 1: Pr>chi2 = 0.035 Wald confidence limits CI: confidence interval * Outcomes per 100 population units

Measures of association strength: The outcome incidence risk among the exposed was 0.4 (95% CI 0.15 to 1.11) times the outcome incidence risk among the unexposed: exposure decreased the outcome incidence risk among the exposed.

The outcome incidence odds among the exposed was 0.23 (95% CI 0.06 to 0.88) times the outcome incidence odds among the unexposed: exposure decreased the outcome incidence odds among the exposed.

Measures of effect in the exposed: Exposure changed the outcome incidence risk in the exposed by -33.91 (95% CI -58.93 to -8.9) per 100 population units. -147% of outcomes in the exposed were attributable to exposure (95% CI -576.7% to 9.9%).

Number needed to treat for benefit (NNTB) and harm (NNTH): Exposure decreased the outcome incidence risk in the exposed. The number needed to treat (expose) to decrease the outcome frequency by one was 3 (95% CI 11 to 2).

Measures of effect in the population: Exposure changed the outcome incidence risk in the population by -4.16 (95% CI -18 to 9.68) per 100 population units. -7.9% of outcomes in the population were attributable to exposure (95% CI -15.4% to -0.9%).

Severe impact of symptoms on everyday life/activities

```
         Outcome +    Outcome -      Total                 Inc risk *
```

Exposed + 15 3 18 83.33 (58.58 to 96.42) Exposed - 41 47 88 46.59 (35.88 to 57.54) Total 56 50 106 52.83 (42.89 to 62.60)

Point estimates and 95% CIs:

Inc risk ratio 1.79 (1.32, 2.43) Inc odds ratio 5.73 (1.55, 21.21) Attrib risk in the exposed * 36.74 (16.62, 56.87) Attrib fraction in the exposed (%) 44.09 (24.19, 58.77) Attrib risk in the population * 6.24 (-7.87, 20.34) Attrib fraction in the population (%) 11.81 (2.97, 19.84) ——————————————————————- Uncorrected chi2 test that OR = 1: chi2(1) = 8.095 Pr>chi2 = 0.004 Fisher exact test that OR = 1: Pr>chi2 = 0.005 Wald confidence limits CI: confidence interval * Outcomes per 100 population units

Symptoms of menopause

```
         Outcome +    Outcome -      Total                 Inc risk *
```

Exposed + 1 8 9 11.11 (0.28 to 48.25) Exposed - 55 42 97 56.70 (46.25 to 66.73) Total 56 50 106 52.83 (42.89 to 62.60)

Point estimates and 95% CIs:

Inc risk ratio 0.20 (0.03, 1.25) Inc odds ratio 0.10 (0.01, 0.79) Attrib risk in the exposed * -45.59 (-68.37, -22.81) Attrib fraction in the exposed (%) -410.31 (-3165.13, 20.24) Attrib risk in the population * -3.87 (-17.57, 9.82) Attrib fraction in the population (%) -7.33 (-13.57, -1.43)
——————————————————————————- Yates corrected chi2 test that OR = 1: chi2(1) = 5.161 Pr>chi2 = 0.023 Fisher exact test that OR = 1: Pr>chi2 = 0.012 Wald confidence limits CI: confidence interval * Outcomes per 100 population units

No problems with performing usual activities

```
         Outcome +    Outcome -      Total                 Inc risk *
```

Exposed + 24 35 59 40.68 (28.07 to 54.25) Exposed - 26 11 37 70.27 (53.02 to 84.13) Total 50 46 96 52.08 (41.64 to 62.39)

Point estimates and 95% CIs:

Inc risk ratio 0.58 (0.40, 0.84) Inc odds ratio 0.29 (0.12, 0.70) Attrib risk in the exposed * -29.59 (-48.93, -10.25) Attrib fraction in the exposed (%) -72.75 (-150.76, -19.01) Attrib risk in the population * -18.19 (-35.98, -0.39) Attrib fraction in the population (%) -34.92 (-62.70, -11.88)
——————————————————————————- Uncorrected chi2 test that OR = 1: chi2(1) = 7.979 Pr>chi2 = 0.005 Fisher exact test that OR = 1: Pr>chi2 = 0.006 Wald confidence limits CI: confidence interval * Outcomes per 100 population units

Extremely expressed anxiety or depression

```
         Outcome +    Outcome -      Total                 Inc risk *
```

Exposed + 7 0 7 100.00 (59.04 to 100.00) Exposed - 43 46 89 48.31 (37.59 to 59.16) Total 50 46 96 52.08 (41.64 to 62.39)

Point estimates and 95% CIs:

Inc risk ratio 2.07 (1.67, 2.57) Inc odds ratio NaN (NaN, NaN) Attrib risk in the exposed * 51.69 (41.30, 62.07) Attrib fraction in the exposed (%) 51.69 (40.10, 61.03) Attrib risk in the population * 3.77 (-10.64, 18.18) Attrib fraction in the population (%) 7.24 (1.53, 12.62)
——————————————————————————- Yates corrected chi2 test that OR = 1: chi2(1) = 5.030 Pr>chi2 = 0.025 Fisher exact test that OR = 1: Pr>chi2 = 0.013 Wald confidence limits CI: confidence interval * Outcomes per 100 population units

Leucocyte-esterase test as “Negative”

```
         Outcome +    Outcome -      Total                 Inc risk *
```

Exposed + 10 2 12 83.33 (51.59 to 97.91) Exposed - 46 48 94 48.94 (38.48 to 59.46) Total 56 50 106 52.83 (42.89 to 62.60)

Point estimates and 95% CIs:

Inc risk ratio 1.70 (1.23, 2.36) Inc odds ratio 5.22 (1.08, 25.11) Attrib risk in the exposed * 34.40 (11.01, 57.78) Attrib fraction in the exposed (%) 41.28 (18.59, 57.64) Attrib risk in the population * 3.89 (-9.98, 17.77) Attrib fraction in the population (%) 7.37 (0.62, 13.66) ——————————————————————- Uncorrected chi2 test that OR = 1: chi2(1) = 5.052 Pr>chi2 = 0.025 Fisher exact test that OR = 1: Pr>chi2 = 0.032 Wald confidence limits CI: confidence interval * Outcomes per 100 population units

Leucocyte-esterase test as "Moderate (2+)"

```
          Outcome +    Outcome -      Total                 Inc risk *
```

Exposed + 10 18 28 35.71 (18.64 to 55.93) Exposed - 46 32 78 58.97 (47.25 to 69.99) Total 56 50 106 52.83 (42.89 to 62.60)

Point estimates and 95% CIs:

Inc risk ratio 0.61 (0.36, 1.03) Inc odds ratio 0.39 (0.16, 0.95) Attrib risk in the exposed * -23.26 (-44.10, -2.42) Attrib fraction in the exposed (%) -65.13 (-180.62, 2.83) Attrib risk in the population * -6.14 (-20.62, 8.33) Attrib fraction in the population (%) -11.63 (-23.57, -0.84) ——————————————————————- Uncorrected chi2 test that OR = 1: chi2(1) = 4.473 Pr>chi2 = 0.034 Fisher exact test that OR = 1: Pr>chi2 = 0.047 Wald confidence limits CI: confidence interval * Outcomes per 100 population units

Pyuria, defined as LEU "Moderate (2+)" and "Large (3+)"

```
          Outcome +    Outcome -      Total                 Inc risk *
```

Exposed + 34 43 77 44.16 (32.84 to 55.93) Exposed - 22 7 29 75.86 (56.46 to 89.70) Total 56 50 106 52.83 (42.89 to 62.60)

Point estimates and 95% CIs:

Inc risk ratio 0.58 (0.42, 0.81) Inc odds ratio 0.25 (0.10, 0.66) Attrib risk in the exposed * -31.71 (-50.83, -12.59) Attrib fraction in the exposed (%) -71.81 (-137.64, -24.21) Attrib risk in the population * -23.03 (-41.28, -4.79) Attrib fraction in the population (%) -43.60 (-75.99, -17.16) ——————————————————————- Uncorrected chi2 test that OR = 1: chi2(1) = 8.498 Pr>chi2 = 0.004 Fisher exact test that OR = 1: Pr>chi2 = 0.004 Wald confidence limits CI: confidence interval * Outcomes per 100 population units

RBC in urine as "Moderate (2+)"

```
          Outcome +    Outcome -      Total                 Inc risk *
```

Exposed + 8 0 8 100.00 (63.06 to 100.00) Exposed - 48 50 98 48.98 (38.74 to 59.28) Total 56 50 106 52.83 (42.89 to 62.60)

Point estimates and 95% CIs:

Inc risk ratio 2.04 (1.67, 2.50) Inc odds ratio NaN (NaN, NaN) Attrib risk in the exposed * 51.02 (41.12, 60.92) Attrib fraction in the exposed (%) 51.02 (40.05, 59.98) Attrib risk in the population * 3.85 (-9.87, 17.57) Attrib fraction in the population (%) 7.29 (1.90, 12.38) ——————————————————————- Yates corrected chi2 test that OR = 1: chi2(1) = 5.814 Pr>chi2 = 0.016 Fisher exact test that OR = 1: Pr>chi2 = 0.006 Wald confidence limits CI: confidence interval * Outcomes per 100 population units

Proteine in urine as "Trace (±)"

```
          Outcome +    Outcome -      Total                 Inc risk *
```

Exposed + 9 1 10 90.00 (55.50 to 99.75) Exposed - 47 49 96 48.96 (38.61 to 59.37) Total 56 50 106 52.83 (42.89 to 62.60)

Point estimates and 95% CIs:

Inc risk ratio 1.84 (1.37, 2.46) Inc odds ratio 9.38 (1.14, 76.96) Attrib risk in the exposed * 41.04 (19.93, 62.15) Attrib fraction in the exposed (%) 45.60 (27.26, 59.32) Attrib risk in the population * 3.87 (-9.92, 17.67) Attrib fraction in the population (%) 7.33 (1.24, 13.04) ——————————————————————- Yates corrected chi2 test that OR = 1: chi2(1) = 4.585 Pr>chi2 = 0.032 Fisher exact test that OR = 1: Pr>chi2 = 0.018 Wald confidence limits CI: confidence interval * Outcomes per 100 population units

Positive urine culture (≥10^3) with Haldane-Anscombe correction for "zero-cells"

```
          Outcome +    Outcome -      Total                 Inc risk *
```

Exposed + 54.5 33.5 88 61.93 (50.96 to 72.08) Exposed - 0.5 5.5 6 8.33 (0.01 to 55.81) Total 55 39 94 58.51 (47.88 to 68.59)

Point estimates and 95% CIs:

Inc risk ratio 7.43 (0.52, 106.13) Inc odds ratio 17.90 (0.96, 334.09) Attrib risk in the exposed * 53.60 (29.27, 77.93) Attrib fraction in the exposed (%) 86.54 (-92.15, 99.06) Attrib risk in the population * 50.18 (25.92, 74.43) Attrib fraction in the population (%) 85.76 (-98.60, 98.98) ——————————————————————- Yates corrected chi2 test that OR = 1: chi2(1) = 4.623 Pr>chi2 = 0.032 Fisher exact test that OR = 1: Pr>chi2 = 0.005 Wald confidence limits CI: confidence interval * Outcomes per 100 population units

Multiple uropathogens

```
          Outcome +    Outcome -      Total                 Inc risk *
```

Exposed + 21 3 24 87.50 (67.64 to 97.34) Exposed - 33 35 68 48.53 (36.22 to 60.97) Total 54 38 92 58.70 (47.95 to 68.87)

Point estimates and 95% CIs:

Inc risk ratio 1.80 (1.35, 2.40) Inc odds ratio 7.42 (2.02, 27.24) Attrib risk in the exposed * 38.97 (21.19, 56.75) Attrib fraction in the exposed (%) 44.54 (26.05, 58.40) Attrib risk in the population * 10.17 (-5.40, 25.73) Attrib fraction in the population (%) 17.32 (6.15, 27.16) ——————————————————————————- Uncorrected chi2 test that OR = 1: chi2(1) = 11.112 Pr>chi2 = <0.001 Fisher exact test that OR = 1: Pr>chi2 = <0.001 Wald confidence limits CI: confidence interval * Outcomes per 100 population units

General non-susceptibility rate of all first uropathogens, to all tested classes of antimicrobial agents

```
         Outcome +    Outcome -      Total                 Inc risk *
```

Exposed + 79 61 140 56.43 (47.80 to 64.78) Exposed - 445 223 668 66.62 (62.90 to 70.19) Total 524 284 808 64.85 (61.45 to 68.15)

Point estimates and 95% CIs:

Inc risk ratio 0.85 (0.73, 0.99) Inc odds ratio 0.65 (0.45, 0.94) Attrib risk in the exposed * -10.19 (-19.15, -1.23) Attrib fraction in the exposed (%) -18.06 (-37.87, -1.09) Attrib risk in the population * -1.77 (-6.63, 3.10) Attrib fraction in the population (%) -2.72 (-5.19, -0.31) ——————————————————————————- Uncorrected chi2 test that OR = 1: chi2(1) = 5.271 Pr>chi2 = 0.022 Fisher exact test that OR = 1: Pr>chi2 = 0.025 Wald confidence limits CI: confidence interval * Outcomes per 100 population units

Non-susceptibility rates of E. coli isolates to 2-nd gen. Cephalosporins

```
         Outcome +    Outcome -      Total                 Inc risk *
```

Exposed + 19 9 28 67.86 (47.65 to 84.12) Exposed - 11 13 24 45.83 (25.55 to 67.18) Total 30 22 52 57.69 (43.20 to 71.27)

Point estimates and 95% CIs:

Inc risk ratio 1.48 (0.89, 2.45) Inc odds ratio 2.49 (0.81, 7.71) Attrib risk in the exposed * 22.02 (-4.37, 48.42) Attrib fraction in the exposed (%) 32.46 (-11.82, 59.20) Attrib risk in the population * 11.86 (-12.18, 35.89) Attrib fraction in the population (%) 20.56 (-9.78, 42.51) ——————————————————————————- Uncorrected chi2 test that OR = 1: chi2(1) = 2.568 Pr>chi2 = 0.109 Fisher exact test that OR = 1: Pr>chi2 = 0.160 Wald confidence limits CI: confidence interval * Outcomes per 100 population units

Forest-plot of RRs of selected parameters

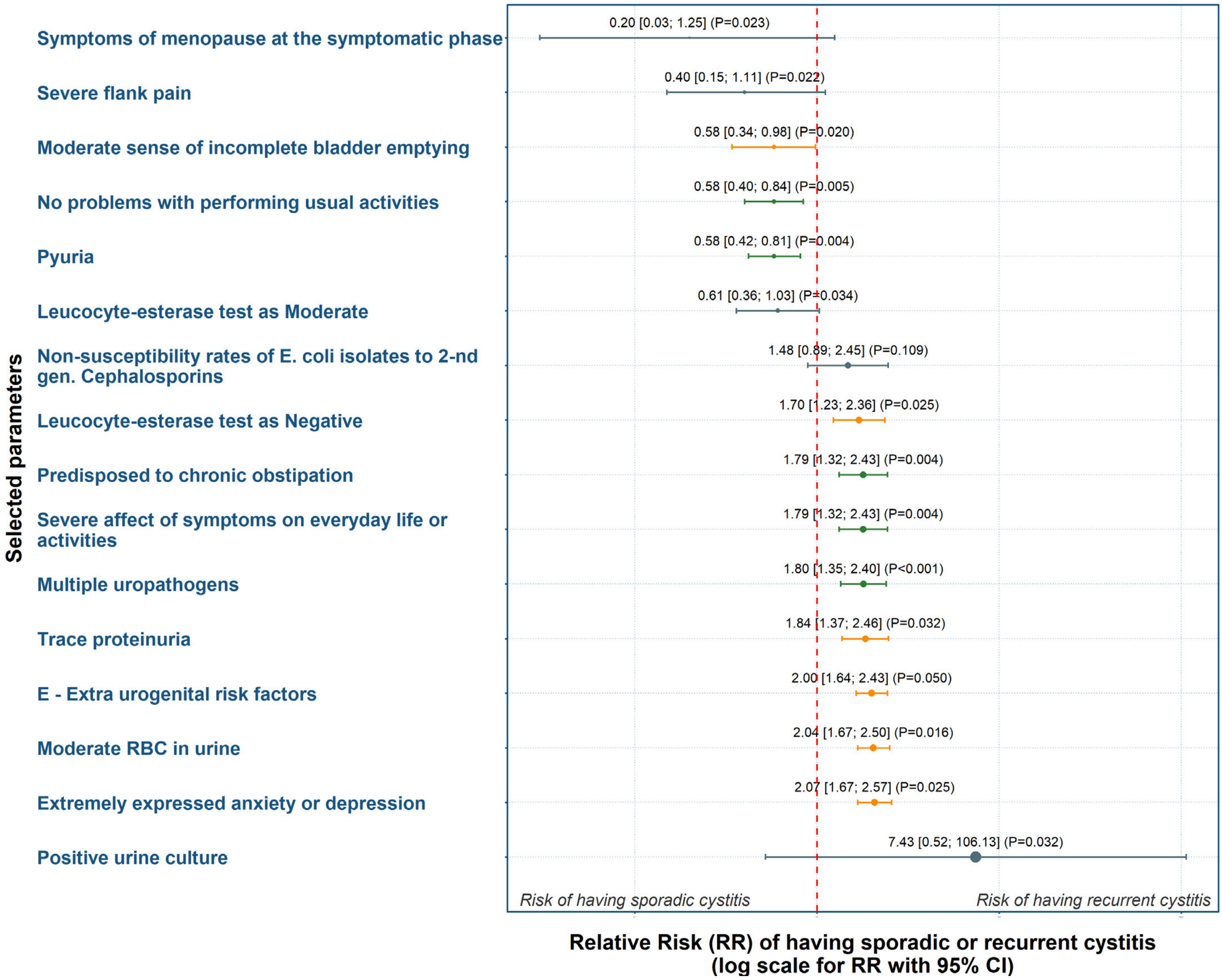

Forest-plot of the Univariate Logistic Regression (ORs) for

the selected parameters

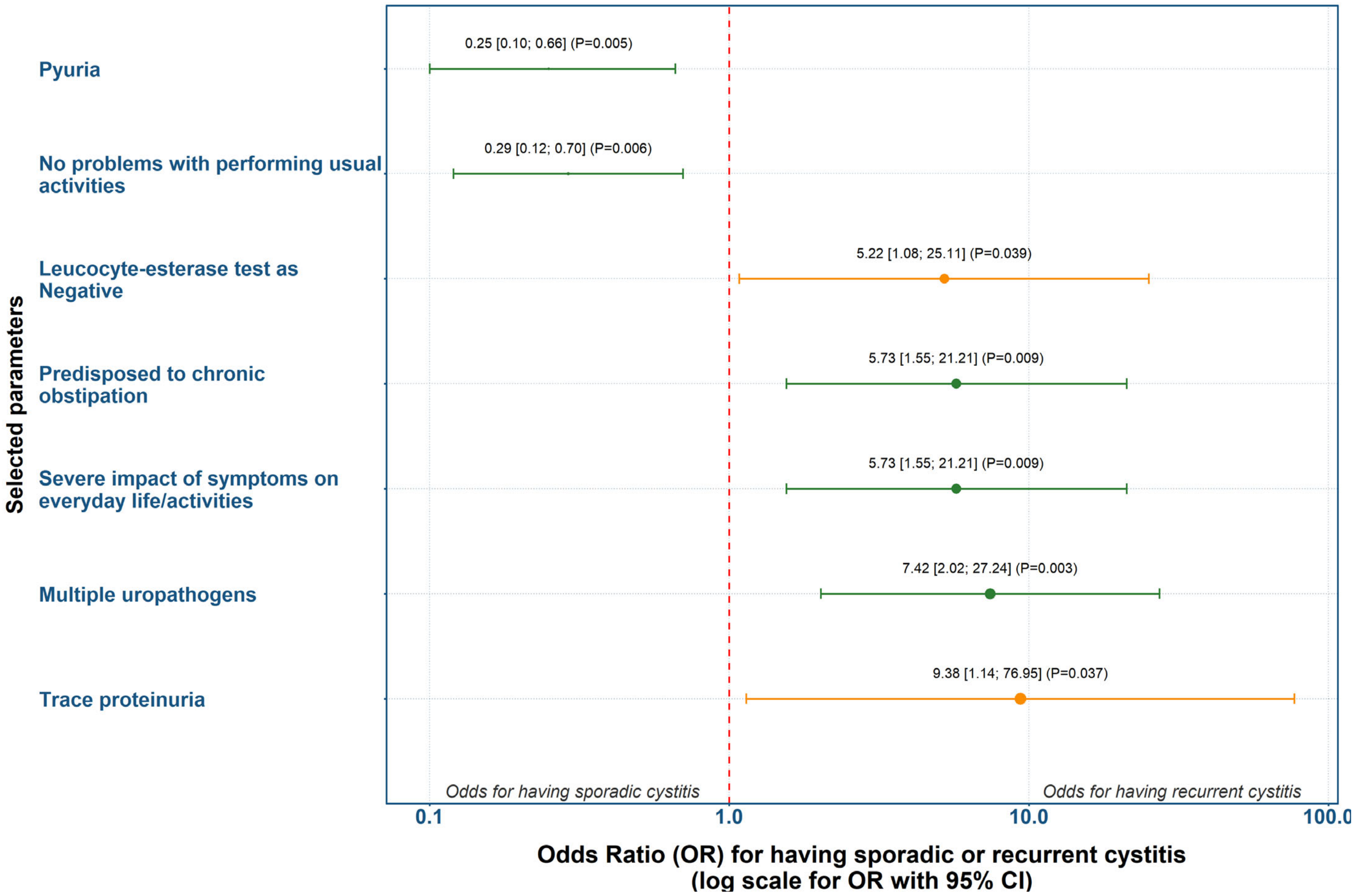

Supplement: Supplementary file 1 [file diagnostics-15-02885-s001.zip › diagnostics-3923247-supplementary.pdf]
